# Establish a PrEP (Pre-Exposure Prophylaxis) Epidemiology, Modeling, and Surveillance (PREMISE) System to Analyze Trends in PrEP Uptake and the Impact of PrEP Programs and Policies: Protocol for a Natural Experiment and Modeling Study in the United States

**DOI:** 10.2196/80911

**Published:** 2026-01-30

**Authors:** Aaron J Siegler, Shi Hao Ernest Koh, Wenting Huang, Eric Hall, Jeb Jones, Courtney R Yarbrough, Xiao Zang, Bohdan Nosyk, Edwin E Corbin-Gutierrez, Patrick S Sullivan

**Affiliations:** 1 Department of Epidemiology Rollins School of Public Health Emory University Atlanta, GA United States; 2 OHSU-PSU School of Public Health Oregon Health & Science University Portland, OR United States; 3 Department of Health Policy and Management Rollins School of Public Health Emory University Atlanta, GA United States; 4 Division of Health Policy and Management School of Public Health University of Minnesota Minneapolis, MN United States; 5 Faculty of Health Sciences Simon Fraser University Burnaby, BC Canada; 6 National Alliance of State and Territorial AIDS Directors Washington, DC United States

**Keywords:** HIV, prevention, pre-exposure prophylaxis, algorithm, modeling, policy

## Abstract

**Background:**

Pre-exposure prophylaxis (PrEP) is highly effective in preventing HIV transmission; yet, many people who would benefit from PrEP are not currently using it. Numerous programs and policies, including those provided under the US Ending the HIV Epidemic effort, have been implemented to increase PrEP use. Programs vary enormously, ranging from telemedicine PrEP support to electronic medical record prompts to social marketing and messaging campaigns. However, limited evidence exists regarding their relative impact on PrEP uptake.

**Objective:**

The aims of the PREMISE (PrEP Epidemiology, Modeling, and Surveillance) research program are to (1) provide context for PrEP scale-up in the United States, (2) assess the impact of different programs and policies on PrEP use, and (3) model the impact of PrEP-related programs and policies on population health.

**Methods:**

The primary outcomes of PrEP use and PrEP-to-need ratios will be extracted from a national medical data aggregator database that represents a majority of PrEP users in the United States. These data will inform all proposed analyses of the project: the dataset will allow the exploration of longitudinal trends in PrEP use by modality as a cohort study, it will be the outcome data for assessing changes associated with particular PrEP programs and policies for quasi-experiments, and it will provide baseline information to inform modeling regarding future impacts of PrEP policies and programs. The implementation of policies will be assessed using legal coding at the state level, and the implementation of programs across health jurisdictions will be assessed using a jurisdiction survey conducted in collaboration with participating health departments. Guided by a legal implementation framework, we will use descriptive and regression analyses to contextualize PrEP scale-up and use quasi-experimental designs to inform causal assessments of the effect of programs and policies. Here, we provide, as preliminary data, our extraction of PrEP prescribing from the national dataset.

**Results:**

This research was funded in August 2024. We obtained the national PrEP database and started data cleaning in March 2025. From 2016 to 2024, there were 20,394,619 claims for medications that were FDA-approved for PrEP, and we determined 13,644,979 claims to be PrEP prescriptions, representing over 1 million PrEP users. For medical benefit claims, there were 34,525 procedure claims for PrEP medications, and we determined 22,910 procedure claims to be for PrEP, representing over 6000 PrEP users.

**Conclusions:**

To optimally use HIV prevention resources, it is critical to understand the effects of different programs and policies. Over 1 million people have started PrEP, and tracking how this scale-up has occurred by PrEP modality and user groups will inform future HIV prevention efforts. By collaborating with health jurisdictions, we will provide systematic data regarding the panoply of programs and policies that have been enacted to support PrEP use.

**International Registered Report Identifier (IRRID):**

DERR1-10.2196/80911

## Introduction

### Background

The US Ending the HIV Epidemic (EHE) launched in 2019 and is led by the US Department of Health and Human Services. EHE sought to reduce new HIV infections and scale up HIV prevention and treatment strategies to improve health outcomes for populations and communities most affected by HIV [1]. A total of 57 jurisdictions across 26 states, as well as Washington, DC, and San Juan, Puerto Rico, have been prioritized in the US EHE initiative [2]. Among these 26 states, 48 counties across 19 states were prioritized due to significant HIV burden, and 7 additional states were prioritized due to substantial occurrence of HIV in rural areas [2].

Pre-exposure prophylaxis (PrEP) is highly effective in preventing HIV transmission and remains the centerpiece of HIV prevention efforts in the United States, yet the scale-up of PrEP has led to stark disparities by race and ethnicity, limiting its impact [3]. PrEP is well tolerated, available in inexpensive generic forms, and prevents over 99% of HIV transmission when taken as directed. In numerous areas, including the United States [4] and New South Wales [5], scale-up of PrEP has been associated with reductions in new HIV transmission at the population level. Despite the spectacular promise of PrEP, less than half of persons in the United States indicated for PrEP were taking it in 2024 [6]. From 2012 to 2017, PrEP use increased by an average of 56% annually [7], followed by a more gradual annual growth of 18% from 2018 to 2021 [3]. The PrEP-to-need ratio (PnR) is a measure of PrEP use that compares the number of PrEP users in a group or geographic area (numerator) to the number of HIV diagnoses (denominator) [8]. This metric has been used to measure PrEP uptake among groups that would most benefit from it in numerous studies and has been used in other areas such as the US Centers for Disease Control and Prevention’s (CDC) recent examination of mpox vaccine provision [9]. PnR assessments have revealed lower PrEP use among Black and Latinx persons, younger persons, women, and persons in the US South relative to epidemic burden [3,10]. Additionally, modeling suggests that the greatest overall HIV epidemic reduction occurs in scenarios with equal PrEP provision relative to HIV burden across racial groups (PnR=1) [11]. Thus, increasing uptake of PrEP in areas with greatest need, as defined by higher HIV incidence, maximizes the number of new infections averted for a given level of PrEP use. National disparities in PrEP provision by race, however, have increased over time [12]. There is an urgent need to identify policies and programs that improve PrEP uptake and PnR.

Despite substantial scale-up of PrEP attributable to past efforts and policies, legal and program environments in which PrEP-eligible people live are highly heterogeneous, and evidence regarding policy impact is lacking. The United States has made significant efforts to scale up HIV PrEP through EHE Programs, with over US $1.5 billion invested in HIV prevention from 2020 to 2024 [13]. EHE funding has been granted to the EHE priority jurisdictions through the Health Resources and Services Administration and CDC mechanisms that allowed state and local health departments and community health centers to implement HIV prevention and treatment initiatives. Diverse programs have been implemented with this funding—ranging from social media messaging to medical records improvements, and from clinician training to telemedicine PrEP prescribing [14-16]—yet, no research program has sought to determine the comparative effectiveness and efficiency of these PrEP interventions on a population basis to guide future investments. Outside of HIV EHE efforts, PrEP uptake may also be influenced by state-level policy decisions and the availability of CDC grant programs such as grants that were designed to support PrEP demonstration projects. Both Medicaid expansion and the use of Pre-Exposure Prophylaxis Drug Assistance Programs (PrEP DAP) offer promising approaches that are associated with increased PrEP use that is also more aligned with epidemic need [17]. It is critical to identify the program elements that have contributed most to successful outcomes within each PrEP service.

National prescription, medical, and diagnosis datasets using data from commercial entities are robust and capture a majority of all US prescriptions with line-level, deidentified individual data [17]. These comprehensive datasets enable accurate, nationwide data on PrEP prescribing, which can be used to develop valuable insights on HIV prevention efforts when analyzed in tandem with data regarding PrEP policies and programs provision. Moreover, such aggregated datasets allow for data to be rapidly assessed regarding the uptake of newer PrEP formulations and modalities (eg, long-acting injectable [LAI] PrEP medications), providing information regarding the use and care characteristics of newer PrEP modalities relative to previously approved modalities.

### Overview of Research Objectives

**Figure 1:**
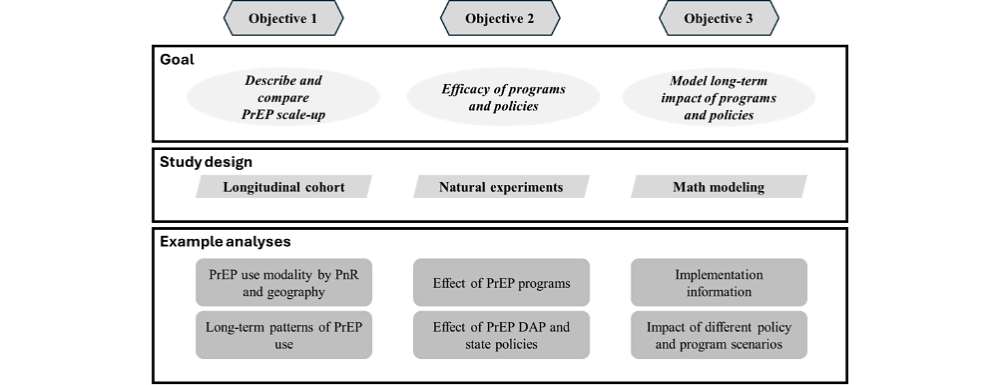
PrEP Epidemiology, Modeling, and Surveillance (PREMISE) research program objectives overview. Overview of the 3 main PREMISE research program objectives, their corresponding study designs, and examples of the planned analyses. PnR: PrEP-to-need ratio; PrEP: pre-exposure prophylaxis; PrEP DAP: Pre-Exposure Prophylaxis Drug Assistance Program.

To support public health jurisdictions in reducing new HIV transmission, the consistent availability of national HIV PrEP data is essential. Such data can be used to describe patterns of PrEP use and to understand which programs and policies are effective at facilitating PrEP uptake, particularly among groups that will most benefit from PrEP use. The PREMISE (PrEP Epidemiology, Modeling, and Surveillance) research program aims to (1) provide context regarding the ways in which PrEP is being brought to scale in the United States, such as the use characteristics of different PrEP modalities; (2) describe the impact of health policies and programs on PrEP use and PnR in the United States; and (3) assess the characteristics of these policies and programs, their impact on population health, and their cost-effectiveness (Figure 1).

## Methods

### Research Setting and Population

The study population consists of patients who had claims between 2016 and 2024 for antiretroviral (ARV) medications approved by the Food and Drug Administration (FDA) for PrEP. Data on PrEP use are available from all 50 states, Washington, DC, and Puerto Rico. Objective 1 will use descriptive and exploratory assessments to contextualize PrEP scale-up. Objective 2 will compare trends in jurisdictions implementing a particular program versus a control group of jurisdictions not implementing that program. Selection of control jurisdictions will vary depending on the level of analysis: for instance, state-level analyses may include all states, whereas analyses at the county level may include counties that are matched by relevant characteristics such as HIV prevalence or county size. Data will be analyzed retrospectively from January 1, 2016, to December 31, 2024, and we will incorporate 4 future annual data updates for the years 2025 to 2028. This study period was selected because the PrEP dataset was considered by the data provider to be internally consistent in the way it was received from pharmacies over the time period, and allows for sufficient time before the launch of EHE programs in 2019. Objective 3 will model program effects for all EHE jurisdictions on future PrEP use based on an up to 20-year time horizon, expanding our team’s existing models for 6 EHE jurisdictions [18,19].

### Research Design Overview

Our analyses will use varied analytic approaches to answer our multiple research questions. To assess changes in PrEP provision over time (objective 1), we will use descriptive and modeling studies. To address gaps in understanding of what programs and policies will best facilitate PrEP scale-up and improved efficiency in the form of PnR (objective 2), we will use quasi-experimental approaches. Many policies and access programs influence PrEP access and uptake among states and over time. Therefore, we will use natural experiments to improve understanding of the impact on PrEP use by collecting information from jurisdictions regarding which programs were conducted and the dates of program operation. We will collect data regarding program characteristics and financial investment to improve our ability to assess causal relationships between different policies or programs and PrEP outcomes. Critical to this design is that, unlike randomized trials, it allows for assessment of health program outcomes with data from programs that were brought to scale outside of research settings by health jurisdictions. Such data will more accurately represent how health interventions are actualized in the United States. Objective 3 will use a mathematical modeling approach that is detailed below. For this paper, we followed the RECORD (Reporting of Studies Conducted Using Observational Routinely Collected Data) checklist (Multimedia Appendix 1) of items to be reported in observational studies using routinely collected health data [20].

### Conceptual Framework

**Figure 2:**
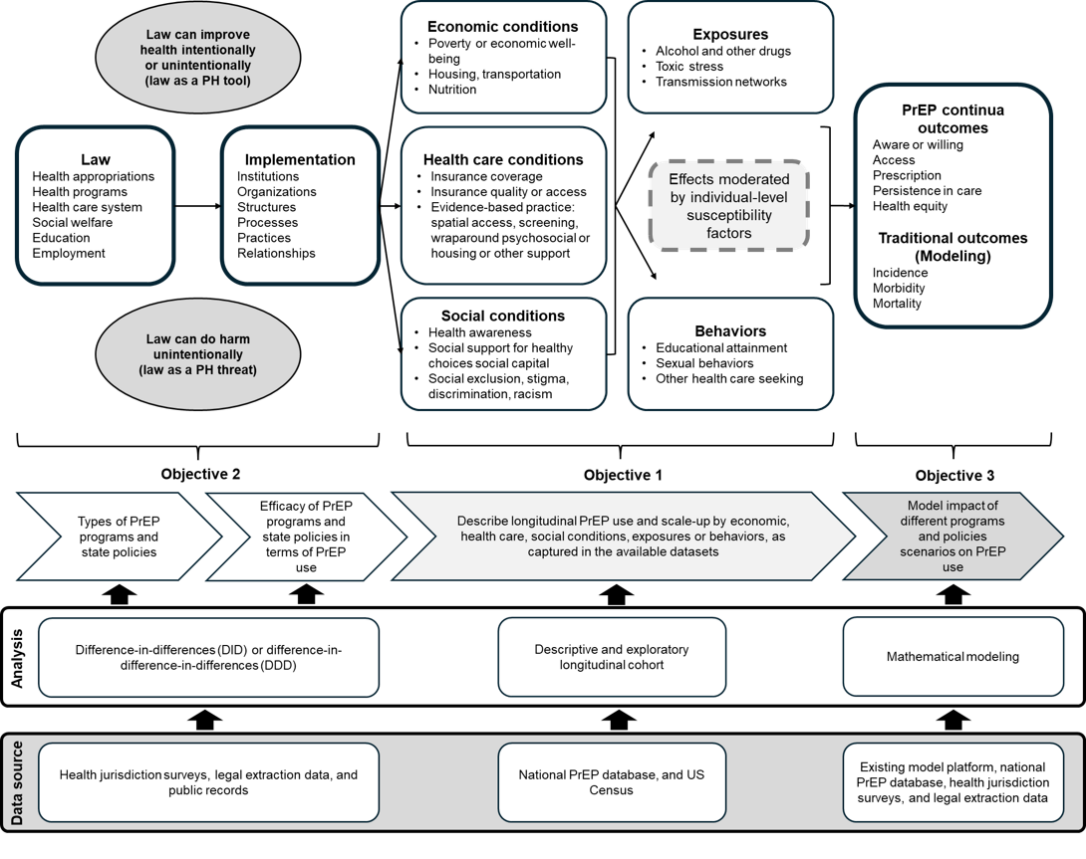
PrEP Epidemiology, Modeling, and Surveillance (PREMISE) research program legal implementation framework. Overview of the legal implementation framework used to guide the PREMISE research program and assess the impact of legal and policy changes on PrEP provision and uptake, and the corresponding data sources and planned analyses. PH: public health; PrEP: pre-exposure prophylaxis.

Our inquiry will be grounded in a legal implementation framework that we have previously proposed to facilitate the development of health policies to optimally address infectious diseases (Figure 2) [21]. In this framework, now adapted to this research program, we posit law as a causal factor influencing HIV transmission. Here, we define law broadly, as sets of rules and regulations that are enforced by the government [22]. As seen in the model, law is enacted through institutions and can influence health positively through constructs such as insurance coverage of an otherwise unaffordable service, or negatively through adverse changes in coverage programs such as Medicaid. Other mechanisms are indirect; for example, wraparound services may assist clients in gaining stable housing, which may in turn facilitate medication adherence. Given this theoretical framework, changes in laws affecting PrEP provision have the potential to have substantial and long-lasting effects on vulnerability to HIV acquisition, including the provision of health services relative to needs for those services. Laws can be either a public health tool (eg, PrEP DAP programs) or a public health threat (eg, judicial rulings that may prevent the US Preventive Service Taskforce recommendations on PrEP from being binding for insurance coverage without cost sharing). Our research acknowledges the more proximal causes of persons taking or adhering to PrEP (eg, an individual’s perceived benefits of taking PrEP) and also focuses on programs and policies that shape and provide context for these decisions. We study programs and policies because (1) they are amenable to change, (2) they are measurable, and (3) there is plausibility that policies and programs can influence PrEP uptake. Relevant data sources will include legal coding and surveys to quantify programs and policies, census data to describe neighborhood factors, and PrEP claims databases to describe use and sociodemographic factors (eg, race, ethnicity, sex, and age). Due to the inherent limitations of our data sources, we cannot measure all components of the framework; instead, we will leverage it to inform the selection of data that are available.

### Data Sources

This program of research will use three main data sources (see Table 1 for a summary of the variables of interest and covariates): (1) data from surveys of health jurisdictions and legal data extracted by the National Alliance of State and Territorial AIDS Directors (NASTAD), (2) a national PrEP database from a third-party data provider (prescription, procedure, and diagnosis claims datasets), and (3) US Census data. Other data sources as they become available will be included in analyses.

**Table 1:** PrEP (pre-exposure prophylaxis) Epidemiology, Modeling, and Surveillance (PREMISE) research program variables and covariates: definitions and data sources^a^.

Construct			Levels or definition		Data source		Definition
**Primary explanatory variables**							
	Program type	Programs supporting PrEP^b^ initiation or retention		Health jurisdiction surveys and legal extraction data		Intervention program implemented	
	Funding spent	Dollars in staffing and direct costs expended to conduct each program		Health jurisdiction surveys		Surrogate for program magnitude	
**Primary outcomes**							
	PrEP starts	New PrEP users in each jurisdiction per time period		National PrEP database		PrEP initiation/population	
	PrEP start needs	New PrEP users, ratio-to-need in jurisdiction per time period		National PrEP database		PrEP initiation/HIV diagnoses	
	PrEP retention	User retained in care per time period		National PrEP database		Average retention time/population	
**Program covariates**							
	Funding	US Centers for Disease Control and Prevention, US Department of Health and Human Services, States		Health jurisdiction surveys and public records		Agency funding the program	
	Jurisdiction	County or state		Health jurisdiction surveys and legal extraction data		Jurisdiction operating the program	
**PrEP user and structural covariates**							
	Age (years)	18-24, 25-39, 40-54, 55 or older^c^		National PrEP database		Age of individual PrEP user	
	Race and ethnicity	Black, Latino or Hispanic, White, Asian, other^c^		National PrEP database		Race and ethnicity of individual PrEP user	
	Gender	Male and female^c^		National PrEP database		Self-identified gender of PrEP user	
	Copay	Average PrEP copay in dollars		National PrEP database		Copay of individual PrEP user	
	PrEP payer	Commercial, government, and other		National PrEP database		Insurer or payer of the PrEP prescription	
	Provider	National Provider Identifier		National PrEP database		Unique identification number of providers	
	Jurisdiction characteristics	% Bachelor’s degree holder, % living in poverty, % uninsured, % Black residents, % Latinx residents		US Census		Proportion of county residents with a characteristic	

^a^Summary of PREMISE research program constructs, including primary explanatory variables, primary outcomes, program covariates, and PrEP user and structural covariates, with corresponding levels or definitions. The table also lists data sources and how each variable is defined for analysis.

^b^PrEP: pre-exposure prophylaxis.

^c^Line-level data acquired from health exchanges. Granular data are not always available such as other identity and additional racial or age categories.

### Health Jurisdiction Survey

We will conduct a survey of health jurisdictions to understand which types of programs were provided in each jurisdiction over time and the degree of resources committed to implementing each program to obtain program data; these data will inform objective 2 analyses. Table 2 details the primary program types that we will assess, representing an array of program settings and types ranging from clinician training to electronic medical records changes to direct financial coverage of PrEP services. We will also assess community change mechanisms such as messaging campaigns and community-based organization-led PrEP outreach. Each of these mechanisms might feasibly impact PrEP use, so generating evidence on the relative impact of each program will be critical to guide future investments. The full text of the jurisdictional survey can be seen in Multimedia Appendix 2. Using lists of health jurisdiction members of NASTAD, we sent the survey to program staff representatives for each of the 50 states and the 8 jurisdictions that receive EHE funding independently from their state. Our instructions clarify that completing the survey may require the knowledge of multiple jurisdictional team members, and the survey is set up to facilitate this with a link that can be accessed by multiple respondents. Data collection launched in May 2025, accompanied by a webinar offered to all health jurisdictions to introduce the survey. We plan to complete data collection in the fall of 2025. To maximize the survey response rate, we will conduct outreach activities, including periodic email reminders, hosting open question and answer sessions, and individual support calls to engage jurisdictions to assist their survey completion.

**Table 2:** PrEP (pre-exposure prophylaxis) Epidemiology, Modeling, and Surveillance (PREMISE) research program health jurisdictions, program types, and details^a^.

Setting and program type			Program details
**In-clinic programs**			
	Training for clinicians and clinical staff	Training or organizational capacity building to increase PrEP^b^ prescribing	
	EHR^c^ optimization	Changes to EHR to ease PrEP delivery: this includes electronic reminders for providers to assess a patient for PrEP eligibility or to develop a list of PrEP candidates using EHR data	
**External clinical programs**			
	Safety-Net PrEP Clinic	Delivery of PrEP through an existing STI^d^ clinic, community health center, pharmacy, or similar public health clinic, aimed at expanding PrEP access among key populations through reduced cost or free services	
	Telemedicine PrEP	Consists of full or hybrid telemedicine PrEP services that reduce or eliminate the need for in-person clinical visits	
	PrEP Assistance Program	Cover the cost of PrEP-related services, either directly or indirectly, usually by reimbursing providers for clinical visits and lab tests, or paying PrEP-related out-of-pocket costs to health plans	
	PrEP navigation services	Provide assistance to find a PrEP provider and address barriers to access PrEP, as well as support strategies to facilitate retention or reinitiation into PrEP care	
**Nonclinical programs**			
	Messaging campaigns	Digital or physical messaging, social marketing aimed at raising awareness about PrEP or sharing information about PrEP services available	
	PrEP outreach and education	Nonclinical outreach and education activities, typically delivered by staff or peers at community-based organizations focusing on raising awareness about PrEP, addressing misinformation, and sharing information about the availability of PrEP	

^a^Health jurisdictions program settings (in-clinic, external clinic, and nonclinical) with corresponding program types and program details. The program types will be assessed to understand which programs were implemented in each jurisdiction over time and the level of resources committed to each to obtain program data.

^b^PrEP: pre-exposure prophylaxis.

^c^EHR: electronic health record.

^d^STI: sexually transmitted infection.

We will also gather information on the passage and implementation of policies regarding PrEP at the state level through a systematic review of legislative records in existing state public health statutes and the legal database Westlaw. Data will be extracted, if available, regarding program types, program start or end dates, funding agencies, jurisdictions implementing the program, the program implementers, and the program financial expenditures. To improve data quality, we will optimize interrater reliability through clear definitions of domains, use raters who are familiar with the content area, and provide thorough training for raters through practice-based learning sessions. Validation of the dataset will be accomplished through conducting parallel extraction of independent coders to determine intercoder agreement for a proportion of the dataset.

### National PrEP Database (Prescription Claim, Procedure Claim, and Diagnosis Code Data)

We have obtained a national PrEP database through a contract with a third-party data provider—a health care company that aggregates national health data from clinics, providers, and pharmacies. This database includes prescription claim data, procedure claim data, and diagnosis claim data. PrEP medications can be billed through either pharmacy or medical benefits, depending on how the medication is dispensed (oral tablet or injection, pharmacy, hospital or clinic) and the requirements of the insurance plan. The diagnosis claim dataset contains medical conditions diagnosed using either the *ICD-9-CM* (*International Classification of Diseases, Ninth Edition, Clinical Modification*) or *ICD-10-CM* (*International Classification of Diseases, Tenth Edition, Clinical Modification*). For each deidentified person included in the database, the following variables are available: prescribed medication, date of each fill, number of pills or injections, sex, age, payer (Medicaid and private health insurance), and ZIP3 (the first 3 digits of a ZIP code). We will seek more specific user locations through both data crosswalks to the county level and providers’ practice location addresses. On June 18, 2025, lenacapavir was approved by the FDA as a twice-yearly LAI PrEP [23], and during the research period, we expect additional medications to be approved by the FDA for PrEP. Therefore, our data provider contract includes access to prescription data for lenacapavir and other potential future modalities.

### US Census Bureau Data

We will gather publicly available data about jurisdictions (eg, characteristics and demographic information) from the US Census Bureau website. Using the Census’s search and filtering functions, we will extract relevant data at the appropriate geographic level (eg, state or county). These data will include distributions of educational attainment, poverty status, insurance coverage, and race and ethnicity composition, each of which will be used to examine jurisdictional differences and their associations with PrEP uptake.

### Other Data Sources

Other potential sources of data may be incorporated into analyses as they become available over time.

#### Primary Outcome Measures

When possible, we will use PrEP outcomes that have been previously defined in prior literature [7,8,17,24-30]. Given a range of analyses across objectives 1-3 that include exploratory, quasi-experimental, and modeling that will require different time horizons and levels of aggregation, we anticipate a number of different PrEP outcomes across analyses for this research program. When appropriate, we will assess PrEP use in terms of starts and retention. PrEP starts will be defined as the number of persons starting PrEP per 100,000 population (PrEP starts per capita). For monthly assessments of this outcome, a PrEP start is defined as a person initiating PrEP for the first time or reinitiating PrEP after a lapse in prescription of a cut point, such as at least 1 month, calculated from the biweekly proportion of days covered (PDC) reaching 0.57 or lower (eg, less than 4 days per week). PDC measures the percentage of days that filled medication prescriptions could provide coverage for a period if the medication is taken as prescribed. The PDC metric has been commonly used in PrEP studies [24,25,27,28,31] and has been suggested as a good metric for studies focusing on PrEP use at specific time points [24]. We will conduct sensitivity analyses to assess the impact of different cut points for lapses in coverage. For retention in care, all PrEP fills not classified as PrEP starts will be considered prescriptions obtained while retained in PrEP care. PrEP retention will be defined as consecutive PrEP prescription refills based on their PrEP modalities, calculated as the time period in continuous PrEP care. For analyses that aggregate beyond the monthly level, such as yearly analyses, we will use definitions previously used in the literature and by the CDC. For yearly analyses, this involves counting any people with a prescribed day of PrEP in that calendar year, including persons with sufficient pills prescribed in the prior year to have days prescribed in the following year [3,8]. We will also analyze how PrEP is used relative to need by assessing the number of persons using PrEP per new HIV diagnosis (PnR). For models in objective 3, the primary outcomes will be HIV infections averted and cost-effectiveness.

#### Data Analyses

We plan to conduct analyses that explore trends in PrEP use over time, reveal the impact of programs and policies, and assess the long-term impact of policies and programs, including health and cost-effectiveness.

To understand PrEP use over time (objective 1), we will include current and new PrEP formulations to explore patterns regarding the growth of PrEP use, changes in retention in PrEP care over time, reinitiation of PrEP over time, and associations with changes in PrEP use. These exploratory assessments will be conducted largely with descriptive and regression analyses, and we anticipate new analyses will emerge as the prevention landscape develops. One area of potential analysis involves characterizing the long-term users of PrEP services, seeking to explore differences between those able to maintain long-term PrEP use versus those with shorter periods of PrEP use. This analysis would likely be largely descriptive and supplemented by regression models. Other analyses may be specific to the PrEP modality. For instance, analyses of injectable PrEP modalities may look at associations between their use and type of insurance coverage, socioeconomic characteristics of users, prior PrEP use patterns, and service provider locations.

To understand the impact of PrEP policies and programs (objective 2), we will leverage the legal coding and health jurisdiction program provision datasets described above to conduct quasi-experiments with outcomes of PrEP starts, PnR, and retention in care. PrEP programs have been adopted by local health authorities in the context of the disbursement of EHE funds as grants to applicants. Despite the lack of random assignment of jurisdictions to policies and programs, we propose a quasi-experimental design that allows us to have findings that reflect several of the characteristics of randomized studies, including temporality and controlling for time-invariant factors. Although quasi-experimental approaches are subject to some limitations inherent to nonrandomized designs such as challenges in controlling for time-varying characteristics, these designs minimize bias relative to other analysis approaches. Moreover, quasi-experimental approaches improve upon the external validity of randomized studies because the data stem from programs that have been enacted in practice. Because of the study design, results will be well positioned to inform real-world programs such as those that comprise EHE efforts. For quasi-experiments, we will conduct analyses using methods such as difference-in-differences (DID) that account for intervention and control jurisdiction trends before program implementation. Because interventions are implemented at different points in time, we will use the heterogeneous DID method proposed by Callaway and Sant’Anna [32] or a similar method to account for potential confounding from multiple implementation periods. Health jurisdictions implementing a program or policy will be the primary intervention unit, and controls will be jurisdictions not implementing a program. DID analyses will be accomplished with regressions that include a term for group (eg, telemedicine PrEP program or not), period (month), and group-by-period interactions that serve to estimate the effect of the intervention. Our approach provides a relatively large sample size for such analyses, with many of the 50 counties or local jurisdictions funded under the EHE program implementing programs under assessment, and other non-EHE jurisdictions also implementing some of the identified programs. Control jurisdictions will be counties or states in the United States that do not implement a particular program, using 9 years of monthly PrEP use data (2016-2024). Depending on characteristics of the intervention, such as statewide provision, we may restrict the control group to jurisdictions meeting certain criteria in order to improve the match between intervention and control groups. For instance, we might match counties by HIV prevalence or jurisdiction size.

Although we have conducted substantial outreach to health jurisdictions to optimize engagement, we anticipate that there will be some missing data from the health jurisdiction survey. It is likely not a valid approach to impute data on whether a program was implemented in a particular jurisdiction. Therefore, analyses will be conducted only among jurisdictions providing data. To explore the impact of the missingness, we will assess descriptive differences between missing and nonmissing jurisdictions for the outcome over time and for key sociodemographic variables. Our outcome assessment will include the determination of policy impacts on PnR, an equity metric. Additionally, we will identify potential inequities in PrEP use based on key geographic, socioeconomic, and demographic characteristics. This may be conducted empirically with interaction terms between the policies of interest and these characteristics. DID models assume that trends in the intervention and control groups are parallel before the intervention. This is a critical consideration because control jurisdictions might have additional interventions to the one under consideration. We will seek to assess the validity of this premise, using techniques such as visual assessments of event-study plots and the Granger causality test to determine whether the design is sufficient to meet the parallel trends assumption. If parallel trends assumptions are not met, we will consider alternative approaches such as multivariable matching, lagged dependent variable regression, and synthetic control models (adapted for multiple treated units). The synthetic control method uses a matching algorithm to create a weighted dataset for the control (nonintervention) group, assigning a weight to each element in the group to eliminate prior differences in critical covariates and the outcome. Another option is to use difference-in-difference-in-differences (DDD) to assess the additional change from a specific policy (eg, telemedicine) above and beyond the overall EHE program effect for funded EHE jurisdictions. The nature of the DID and DDD designs controls for confounders that do not vary over time. Therefore, to optimize model efficiency, we will not control for variables expected to remain constant over time (eg, area-level poverty), only adding time-variant covariates. To further ascertain the validity of the DID approach, we will conduct placebo tests for policies that our models have found to have statistically significant effects on PrEP use. These tests assign earlier implementation dates selected at random (eg, false) to treated units to observe if we continue to reject the null hypothesis under these “false” implementation dates. Statistically insignificant results from the placebo tests will increase confidence that the model outcomes are valid. Further, we will assess the sensitivity of our models to the implementation time period by conducting the Bacon-Goodman [33] decomposition and reporting any disproportionate influence of certain implementation periods. We will seek to conduct additional sensitivity analyses that explore whether funding levels determine the impact of programs, with the program intervention variable being related to prevention budget expended rather than whether the program was enacted. Analyses will be conducted in SAS 9.4, STATA 19, or similar software. Given that there are multiple policies, it will be critical to address challenges with multiple comparisons. To accomplish this, we will, where possible, seek unified model approaches that simultaneously test the effects of multiple programs.

To assess the impact of scaling-up different PrEP programs and policies investigated in health jurisdictions (objective 3), we will use a previously published mathematical model leveraging the estimates of the effectiveness and cost of each policy and program from DID analyses to inform our models. The Localized Economic Modeling (LEM) platform is a dynamic compartmental HIV transmission model used to simulate changes in HIV epidemics. Populated with locally sourced data on demographic, epidemiological, and policy conditions for specific jurisdictions, the model has been previously applied to evaluate combination implementation strategies for EHE in 6 US cities: Atlanta (Georgia), Baltimore (Maryland), Los Angeles (California), Miami (Florida), New York City (New York), and Seattle (Washington) [18,19,34-37]. The model stratifies the study population by biological sex, race and ethnicity (non-Hispanic Black, Hispanic or Latino, non-Hispanic White, and other), HIV transmission risk group (men who have sex with men, people who inject drugs, and heterosexuals), and sexual risk behavior intensity (high- or low-risk). The adult population is followed from susceptibility to HIV infection through seroconversion, diagnosis, and treatment with antiretroviral therapy (ART), accounting for observed disparities in access to HIV health services, including HIV testing, ART, PrEP, and harm reduction services. The LEM has been applied to evaluate various public health interventions related to EHE and their health impacts [36,38,39]. As part of ongoing efforts, we are expanding and updating the underlying evidence while adapting and recalibrating the model for all 48 EHE priority counties and Washington, DC (not including San Juan, Puerto Rico, and the 7 EHE priority states with high HIV incidence in rural areas). The updated models will be leveraged to examine incident HIV infections averted, incremental costs, gains in quality-adjusted life years (a composite measure capturing HIV-related morbidity, mortality, and transmission) [40], cost-effectiveness, and health impacts (measured by disparities in HIV incidence) of the effective PrEP scale-up programs or policies identified in objective 2—compared to a “status quo” scenario assuming no further efforts to scale-up access to PrEP. We will perform the modeling analysis of PrEP programs and policies up to 20-year time horizon, evaluating them both individually and in combination to capture their long-term health and economic impacts in different jurisdictions, as well as potential synergistic effects between programs and policies. Analyses will be conducted according to best practices guidelines in simulation modeling [41] and cost-effectiveness analysis [42].

#### Power Calculation: Objective 2

The study design achieves 90% power to detect a minimum difference between intervention and control county PrEP rate means of 2.4 PrEP starts per 100,000 population. This is based on a sample size of 8,784,000 county-person-month observations, a figure obtained from a minimum estimate of 6 counties implementing a particular program and 238 control counties not implementing the program. We assume that each county has an average of 300 individual PrEP users (a conservative estimate because the 2021 average and median county PrEP users are 1210 and 482), and we assume an average of 60 months of follow-up (a conservative estimate because the total follow-up period is 144 months). The standard deviation of PrEP use at the county level is 73, and we conservatively assumed a small (0.05) within-person correlation. The power assessment is based on a time-by-treatment interaction test using a mixed-model analysis with a significance of 0.05. We performed a similar analysis for state-level policies and found a minimum detectable difference of <3 additional PrEP starts. Power calculations were performed in PASS 2022.

#### Preliminary Findings Regarding PrEP Use Determination

**Figure 3:**
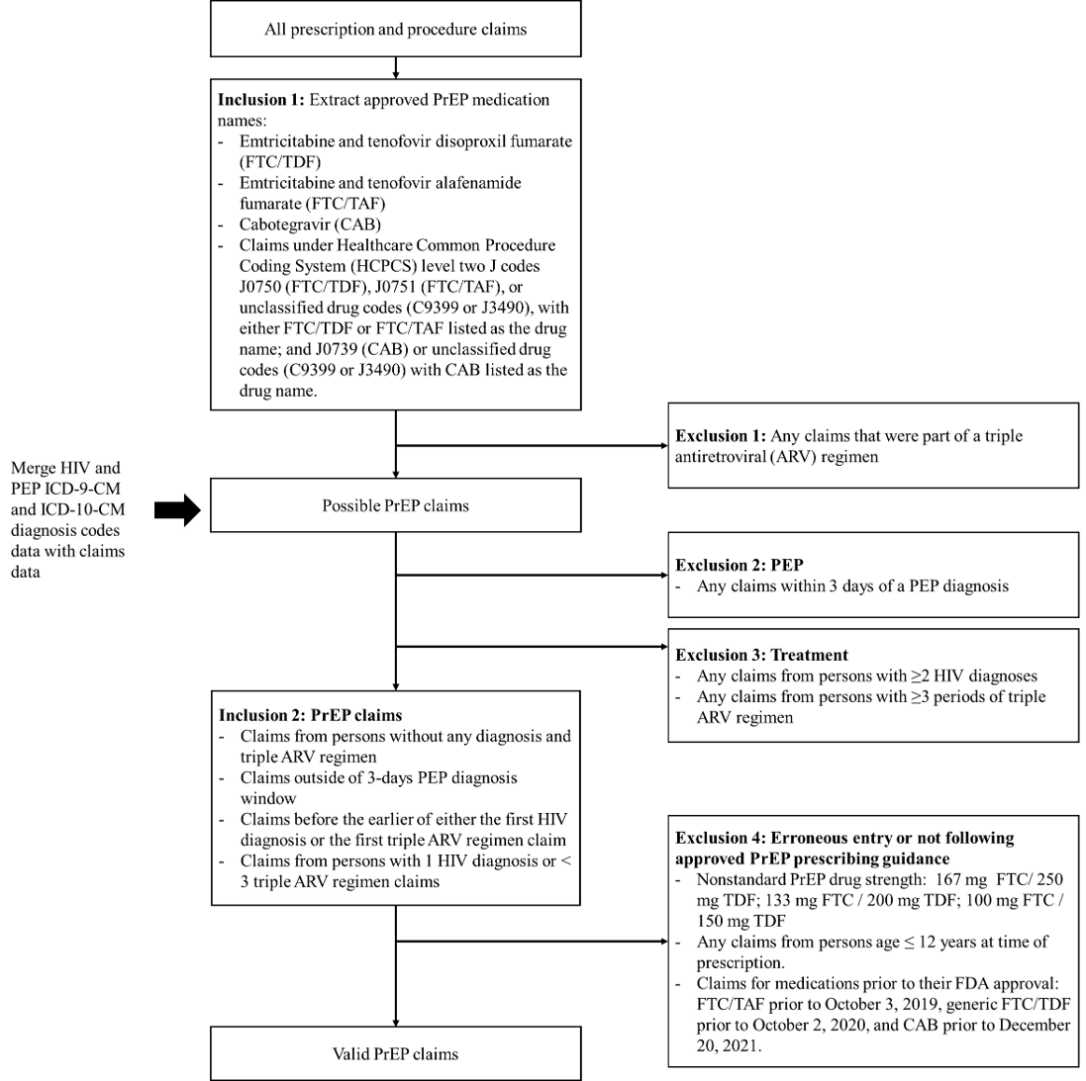
PrEP Epidemiology, Modeling, and Surveillance (PREMISE) research program of PrEP use determination algorithm flow diagram. The flow diagram shows the algorithm developed to identify eligible PrEP prescription and procedure claims from the national PrEP database from a third-party data provider, including the inclusion and exclusion criteria applied at each decision point, leading to the final PrEP prescription and procedure datasets. ARV: antiretroviral; CAB: cabotegravir; FDA: Food and Drug Administration; FTC: fluoro-thiacytidine; ICD-9-CM: International Classification of Diseases, Ninth Revision, Clinical Modification; ICD-10-CM: International Classification of Diseases, Tenth Revision, Clinical Modification; PEP: postexposure prophylaxis; PrEP: pre-exposure prophylaxis; TAF: tenofovir alafenamide fumarate; TDF: tenofovir disoproxil fumarate.

We sought to determine levels of PrEP use in the third-party provider dataset (Symphony Health, an ICON plc Company, PatientSource®, January 1, 2016 to December 31, 2024). For prescriptions from aggregated datasets, a medication may sometimes be used for more than one purpose, so it is necessary to determine which prescriptions are intended for PrEP use. Informed by prior work in this area [43], we developed an algorithm to identify prescriptions for PrEP use (see Figure 3), with our algorithm foremost guided by published clinical guidance [44-50] and the availability of relevant data in the dataset. For this analysis, we extracted all prescription claims with approved PrEP medication names, that is, emtricitabine and tenofovir disoproxil fumarate (fluoro-thiacytidine [FTC]/TDF), emtricitabine and tenofovir alafenamide fumarate (FTC/TAF), and cabotegravir (CAB) (inclusion criteria 1), and then excluded the claims associated with either HIV treatment (ART) or postexposure prophylaxis (PEP). We first excluded any prescription claims that were part of a triple ARV regimen (exclusion criterion 1) and then excluded any prescription and procedure claims within 3 days of a PEP diagnosis (exclusion criterion 2). To further exclude prescription claims that were intended for HIV treatment, we incorporated relevant diagnosis data, a dataset of over 7 million HIV diagnosis claims and 60,000 PEP diagnosis claims. Medical conditions diagnosed by providers were provided in the dataset with *ICD-9-CM* or *ICD-10-CM* codes. While *ICD-10-CM* codes have replaced the *ICD-9-CM* codes since 2015 for billing purposes, some health systems may still be using *ICD-9-CM* codes. We extracted all codes related to HIV conditions (see Multimedia Appendix 3) and aggregated the number of HIV diagnoses based on the number of HIV condition-related codes for each patient. Given that for PrEP or PEP care HIV diagnoses can be mistakenly entered into the medical chart [51-53], we did not automatically exclude all persons with a single HIV diagnosis. Instead, we excluded (1) all claims for persons with two or more HIV diagnoses, (2) claims for persons with one HIV diagnosis and three or more periods of triple ARV regimen, and (3) claims for persons with no HIV diagnosis but with three or more periods of triple ARV regimen that indicate likely treatment (exclusion criterion 3). For persons with only a single HIV diagnosis and 2 or fewer triple ARV prescriptions, the triple ARV prescriptions could be for PEP, so nontriple therapy PrEP medication claims for these persons were eligible for inclusion. For individuals meeting exclusion criterion 3, the HIV diagnosis criteria, any claims for PrEP coming before the earlier of either the first HIV diagnosis or the first triple ARV regimen claim were not excluded (inclusion criteria 2). Last, we excluded the small number of claims that could represent entry errors or did not follow prescribing guidance (exclusion criterion 4): any claims for PrEP that did not reflect the standard drug strength, any claims for persons under the age of 12 years at the time of prescription, and claims for medications prior to their FDA approval—FTC/TAF prior to October 3, 2019, generic FTC/TDF prior to October 2, 2020, and CAB prior to December 20, 2021.

PrEP medications may be billed through either pharmacy or medical benefits, depending on how the medication is dispensed and the requirements of the insurance plan. Oral PrEP is typically filled at a pharmacy, and so it is captured under pharmacy claims within national aggregator datasets. In rare cases, oral PrEP can also be filled through medical benefits under the Healthcare Common Procedure Coding System (HCPCS) level two J codes, such as J0750 (FTC/TDF), J0751 (FTC/TAF), or unclassified drug codes (C9399 or J3490), with either FTC/TDF or FTC/TAF listed as the drug administered. LAI PrEP (ie, CAB) is administered in clinical settings and therefore reimbursed through medical benefits, which are captured as procedure claims using the HCPCS level two J codes such as J0739 (CAB), or unclassified drug codes (C9399 or J3490) with CAB listed as the drug administered. Depending on insurance coverage, LAI PrEP may be obtained via specialty pharmacies and covered under the pharmacy benefit. Although there is limited overlap of these datasets, we sought to ensure deduplication across PrEP claims across prescription and procedure claims datasets. For LAI PrEP, prescription and procedure claims occurring within 15 days of each other were considered as the same injection and deduplicated. A 15-day period was selected because it is less than the minimum clinical window of 1-month spacing for LAI PrEP [54]. For oral PrEP, prescription and procedure claims occurring on the same day or within a 3-day window were considered as the same prescribing event and deduplicated accordingly. We selected a 3-day window because some monthly patients can opt to refill their medications early, but we expect a small period of time to elapse before they refill.

### Ethical Considerations

This study involves secondary data analysis of fully deidentified prescription claims, procedure claims, and diagnosis code data, as well as a survey of US health jurisdictions to gather information on PrEP-related funding and program implementation. There was no human subjects enrollment or interaction, and no collection or use of any personal identifiable information. As such, this study does not constitute human subjects research or clinical investigation as defined in the federal regulations and was not subjected to institutional review board (IRB) review [55]. Investigators completed a worksheet provided by the Emory IRB, documenting that IRB review was not required. The study claims data are accessed by Emory under a contract and data use agreement with a third-party provider that specifies appropriate storage and data security procedures. As is normative for this type of claims data, the contract does not allow for external sharing of line-level data by Emory. However, other investigators can access the data directly through the data provider, ICON plc.

## Results

### Preliminary Findings

**Figure 4:**
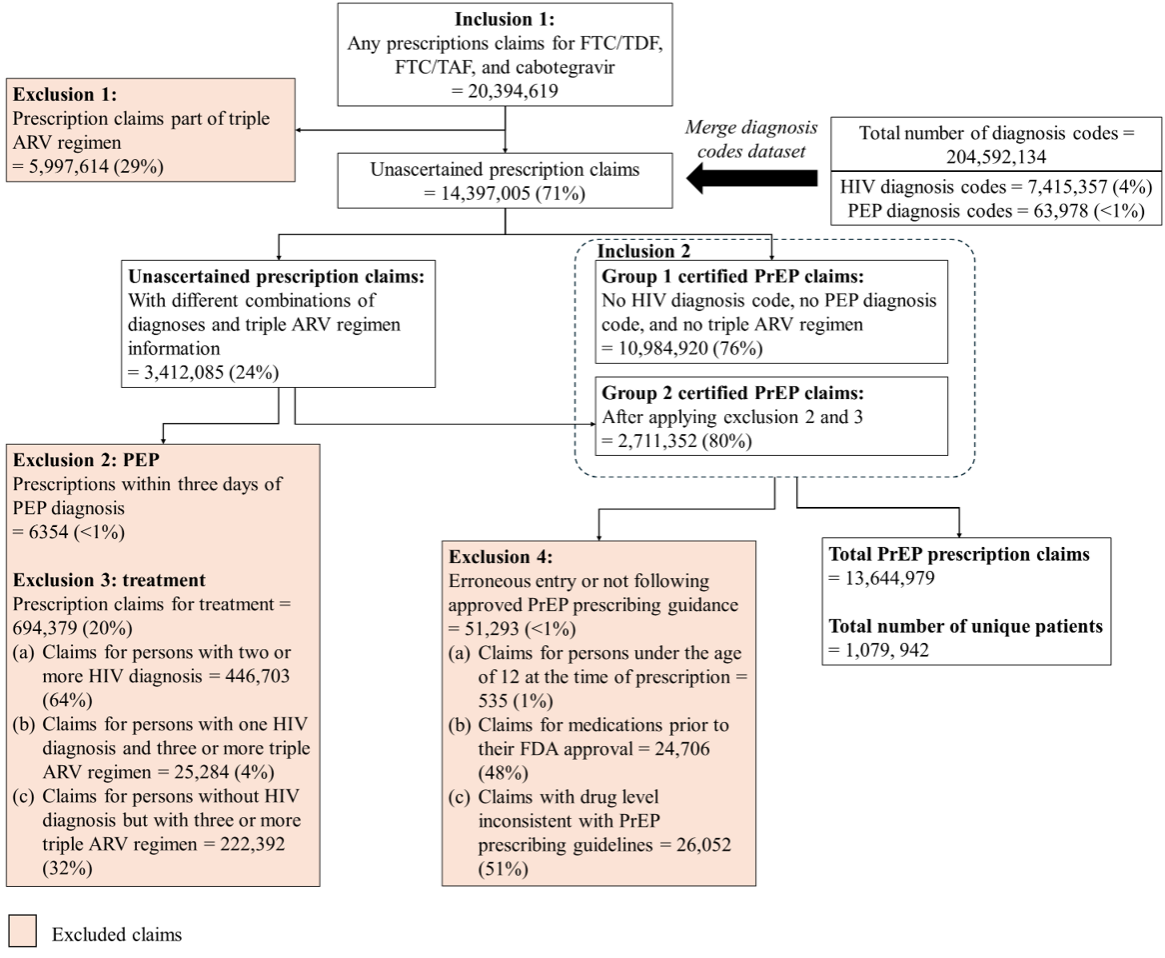
PrEP Epidemiology, Modeling, and Surveillance (PREMISE) research program filtering process for identifying and extracting valid PrEP prescription claims. This figure illustrates the determination of PrEP prescriptions; after initial analysis of prescription claims, diagnosis claims inform exclusion, allowing us to identify the total number of PrEP prescription claims and patients. From 2016 to 2024, there were 20,394,619 prescription claims for FTC/TDF, FTC/TAF, and cabotegravir. We excluded 5,997,614 prescription claims identified to be part of the triple ARV regimen (exclusion 1), 6354 prescription claims occurring within 3 days of PEP diagnosis identified to be for PEP (exclusion 2), 694,379 prescription claims identified to be for treatment (exclusion 3), and 51,293 prescription claims identified to be erroneous entries or not following approved PrEP prescribing guidance (exclusion 4). The final prescription dataset contained 13,644,979 PrEP prescription claims representing 1,079,942 unique patients. ARV: antiretroviral; FTC: fluoro-thiacytidine; PEP: postexposure prophylaxis; PrEP: pre-exposure prophylaxis; TAF: tenofovir alafenamide fumarate; TDF: tenofovir disoproxil fumarate.

From 2016 to 2024, there were more than 20 million claims of medications approved by the FDA for PrEP: FTC/TDF, FTC/TAF, and CAB (see Figure 4). From these prescriptions, we excluded about 6 million claims for coinciding periods that included triple ARV drug regimens (exclusion criterion 1). We then merged the diagnosis claims into the dataset to allow for further determination. We confirmed 11 million PrEP prescription claims that were connected to individuals who had no HIV diagnosis, no PEP diagnosis, and had never used a triple ARV regimen (Group 1 certified PrEP claims). For the remaining 3 million claims, we used clinical guidance for possible other uses of the drugs to determine whether a prescription was for PrEP (Group 2 certified PrEP claims) or for an alternative use. We determined 700,000 of these claims to be not for PrEP use: 6000 claims for PrEP medications that occurred within 3 days of a PEP diagnosis (exclusion criterion 2), 447,000 claims from individuals with 2 or more HIV diagnosis codes (exclusion criterion 3), 247,000 claims from individuals with only one HIV diagnosis, and 3 or more claims for triple ARV regimens (exclusion criterion 3). About 25,000 claims for PrEP medications occurred prior to FDA medication approval, 500 claims for individuals under the age of 12 years at the time of prescription, and 26,000 claims did not reflect standard PrEP drug strengths (exclusion criterion 4). This led to a final prescription dataset with over 1 million cumulative patients having initiated PrEP and over 13 million separate PrEP prescriptions.

**Figure 5:**
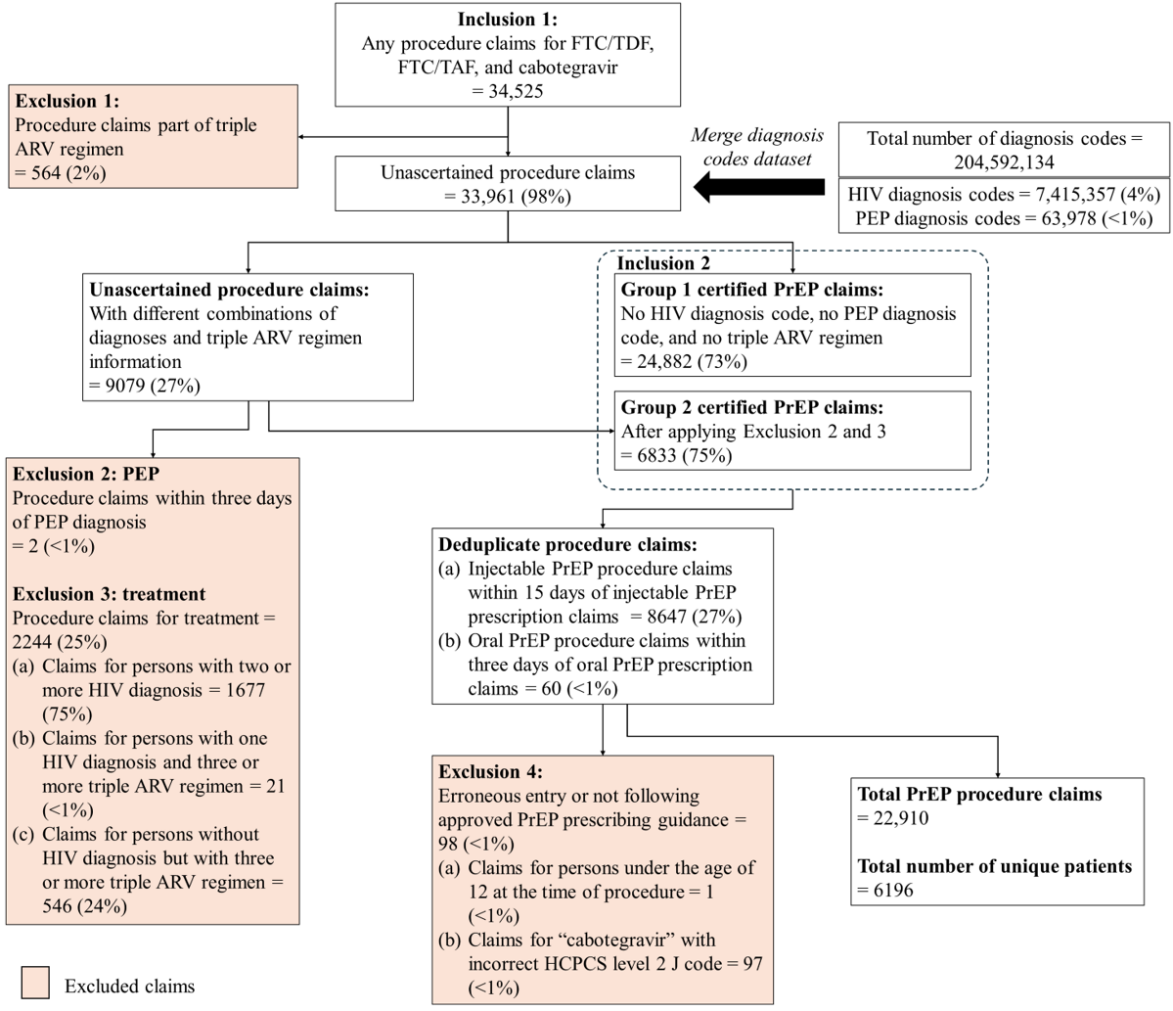
PrEP Epidemiology, Modeling, and Surveillance (PREMISE) research program filtering process for identifying and extracting valid PrEP procedure claims. This figure illustrates the process of determining PrEP procedures from the dataset, following the exclusion criteria as described in the main text. After initial analysis with procedure claims, we add diagnosis claims to further exclude procedures anticipated to not be for PrEP, showing the final number of PrEP procedure claims and patients retained. From 2016 to 2024, there were 34,525 procedure claims for FTC/TDF, FTC/TAF, and cabotegravir. We excluded 564 procedure claims identified to be part of the triple ARV regimen (exclusion 1), 2 procedure claims occurring within 3 days of PEP diagnosis identified to be for PEP (exclusion 2), 2244 procedure claims identified to be for treatment (exclusion 3), and 98 procedure claims identified to be erroneous entries or not following approved PrEP prescribing guidance (exclusion 4). The final procedure dataset contained 22,910 PrEP procedure claims representing 6196 unique patients. ARV: antiretroviral; FTC: fluoro-thiacytidine; HCPCS: Healthcare Common Procedure Coding System; PEP: postexposure prophylaxis; PrEP: pre-exposure prophylaxis; TAF: tenofovir alafenamide fumarate; TDF: tenofovir disoproxil fumarate.

For medical benefits claims, there were about 34,000 procedure claims for PrEP provided in a clinical setting (see Figure 5). From these procedure claims, we excluded about 600 claims for coinciding periods that included triple ARV drug regimens (exclusion criterion 1). Using the same diagnosis claims dataset as described above, we confirmed 25,000 PrEP procedure claims for individuals who had no HIV diagnosis, had no PEP diagnosis, and had never used a triple ARV regimen (Group 1 certified PrEP claims). For the remaining 9000 claims, using the same clinical guidance as described above to determine whether a procedure was for PrEP (Group 2 certified PrEP claims), we determined 2200 of these claims to be not for PrEP use: 1670 claims from individuals with 2 or more HIV diagnosis codes (exclusion criterion 3), with the remaining claims from individuals with only 1 HIV diagnosis and 3 or more claims for triple ARV regimens (exclusion criterion 3), or PrEP medications that occurred within 3 days of a PEP diagnosis (exclusion criterion 2). About 9000 procedure claims for PrEP were duplicates. Under exclusion criterion 4, there was 1 claim for an individual under the age of 12 years at the time of procedure and about 100 claims for LAI PrEP with the drug name “cabotegravir” listed but an incorrect HCPCS level two J code. This led to a final procedure dataset with over 6000 cumulative patients having initiated PrEP through medical benefits with about 22,500 procedure claims for LAI PrEP and about 400 procedure claims for oral PrEP.

By developing an algorithm to determine PrEP use from other prescribing of ARV medication, this early analysis provides outcome data for all subsequent components of the PREMISE project. For objective 1, these data will be used to inform our descriptive and longitudinal analyses of changes in PrEP use over time. To accomplish objective 2, the data will be used as an outcome variable, allowing for the determination of the impact of PrEP policies and programs. To guide objective 3, these data will be used to inform the modeling of the future impacts and cost-effectiveness of different programs and policies.

### Anticipated Research Timeline

Extraction of PrEP prescription and procedure claims was completed as part of objective 1 in 2025. Following this critical component, the planned analyses listed under objectives 1-3 in Table 3 will be conducted. This timeline provides detailed information about the planned analyses for the grant, although we may add additional analyses as new products (eg, long-acting lenacapavir) come to market or new programs, policies, or their cessation occur over time.

**Table 3:** PrEP (pre-exposure prophylaxis) Epidemiology, Modeling, and Surveillance (PREMISE) anticipated timeline and research activities across the 5-year grant period^a^.

Activities			Year																					Data source			Planned analyses
			1				2				3				4				5								
			<>		<>		<>		<>		<>		<>		<>		<>		<>		<>						
**Objective 1: Longitudinal PrEP^b^ provision analyses**																											
	Descriptive analyses of PrEP modality (TDF^c^, TAF^d^, and LAI^e^) by race	✓		✓																		National PrEP database			Descriptive and exploratory longitudinal cohort		
	10-year longitudinal patterns of PrEP use					✓		✓														National PrEP database			Descriptive and exploratory longitudinal cohort		
	Analyses of LAI-PrEP users and retention in care					✓		✓														National PrEP database			Descriptive and exploratory longitudinal cohort		
**Objective 2: Quasi-experiments of PrEP policies and programs**																											
	Develop program and policy dataset	✓		✓		✓																Health jurisdiction surveys, public records, and legal extraction data			DID^f^ or DDD^g^		
	Conduct quasi-experiments on PrEP initiation: EHE^h^, PrEP DAP^i^, Medicaid Expansion, and pharmacist-provided PrEP					✓		✓		✓		✓		✓								National PrEP database, program, and policy dataset			DID or DDD		
	Assess policy effects on PrEP initiation: PrEP DAP and Medicaid					✓		✓		✓		✓										National PrEP database, program, and policy dataset			DID or DDD		
	Assess program and policy effects on retention in PrEP care					✓		✓		✓		✓		✓								National PrEP database, program, and policy dataset			DID or DDD		
**Objective 3: Model epidemic impact and cost-effectiveness analyses**																											
	Modeling program and policy long-term impact									✓		✓		✓		✓						National PrEP database, program, and policy dataset			Mathematical modeling		
	Cost-effectiveness analyses											✓		✓		✓		✓				National PrEP database, program, and policy dataset			Mathematical modeling		
**Dissemination phase**																											
	AIDSVu blog posts and website infographics			✓		✓		✓		✓		✓		✓		✓		✓		✓		National PrEP database, program, and policy dataset			N/A^j^		

^a^This table outlines the planned research activities for the 5-year grant period, detailing the research objectives, corresponding analyses, required data sources, and the anticipated year in which each activity will be conducted.

^b^PrEP: pre-exposure prophylaxis.

^c^TDF: tenofovir disoproxil fumarate.

^d^TAF: tenofovir alafenamide fumarate.

^e^LAI: long-acting injectable.

^f^DID: difference-in-differences.

^g^DDD: difference-in-difference-in-differences.

^h^EHE: Ending the HIV Epidemic.

^i^PrEP DAP: Pre-Exposure Prophylaxis Drug Assistance Program.

^j^N/A: not applicable.

## Discussion

### Overview

To reach EHE targets, it is crucial to have consistent national HIV PrEP data to describe patterns of use and to understand which programs and policies are effective at facilitating PrEP uptake. In PREMISE, we propose to build on our history of developing public PrEP data resources and innovative metrics of PrEP use. The research program comes from a collaborative and interdisciplinary group that will use national PrEP data, legal coding, and policy coding to inform the US HIV epidemic response. Prior studies internationally assessing PrEP policies have largely focused on whether PrEP is legally accessible and covered by insurance or public programs [56,57], which may not fully reflect the effectiveness of PrEP implementation. There is a need to understand the impact of policies and programs that support and promote PrEP as a public health good.

### Limitations

The aggregated medical claims dataset that provides PrEP use outcome data for all study aims contains a majority of claims in the United States but is subject to a number of limitations. First, it does not include prescriptions from closed health care systems, including the Veterans Affairs. Data must therefore be interpreted with caution for areas with high numbers of persons enrolled in closed health care systems, such as Kaiser Permanente users. Second, PrEP use in clinical trials and other informal channels may not be captured by the claims dataset. A limitation of the quasi-experimental approach is the lack of randomization to determine the effects of programs and policies, a limitation inherent to such assessments. We will minimize the impact of this limitation by using analytic designs that carefully adjust for secular trends and will also explore in our findings other considerations for establishing a causal relationship, including effect size, dose-response, and plausibility of the causal mechanism (eg, some programs might be anticipated to have a higher impact on care initiation than retention in care). Another limitation of the quasi-experimental approach is that the COVID-19 pandemic impacted PrEP use during the epidemic period [58-60]; this could influence the outcomes of analyses. To address this challenge, we will conduct a series of visual and quantitative sensitivity analyses for each program or policy that considers the pandemic period and its impact on results. There is a dependency in identifying programs with positive effects and modeling of these effects. We anticipate that some of the funds invested in EHE will have a quantifiable impact on PrEP use and care retention, but if we find no impact from any programs, we will conduct modeling informed by other data, such as those from clinical trials and observational studies. Another limitation is that funding for EHE programs could be discontinued, but based on our power calculations, we still will have sufficient data to perform the program and policy analyses, and evidence of success may become critical to encouraging funding reauthorization. Another potential challenge is the possibility of new medication approvals; we have discussed this with our aggregated data provider, and the contract will include all current and future PrEP medication data. Lastly, while the general structure of the algorithm may be applicable to other datasets, it may not be fully transferable due to potential variations in data structure, coding practices, and available variables across different data providers.

### Conclusions

HIV PrEP has the potential to contribute substantially to ending the HIV epidemic, and increasing PrEP coverage to persons at substantial risk of transmission is critical to deliver on the promise of the intervention. Diverse programs have been launched to support increased PrEP use and retention in care, and systematic data are needed to understand the impact of these programs in real-world implementation settings such as health departments. The analyses proposed in this protocol will fill this gap, providing critical information regarding program impact. We anticipate that such data will facilitate increased investment in prevention efforts by providing an evidence base to understand the relative and absolute impacts of PrEP promotion interventions.

## Appendix

Multimedia Appendix 1 RECORD checklist.

Multimedia Appendix 2 PrEP impact evaluation survey that was developed and used to survey health jurisdictions to understand the types of PrEP programs that were provided in each jurisdiction over time and the degree of resources committed to implementing each program to obtain program data. PrEP DAP: Pre-Exposure Prophylaxis Drug Assistance Program.

Multimedia Appendix 3 ICD-9-CM and ICD-10-CM codes related to HIV conditions. ICD-9-CM: International Classification of Diseases, Ninth Revision, Clinical Modification; ICD-10-CM: International Classification of Diseases, Tenth Revision, Clinical Modification.

## Abbreviations

ART: antiretroviral therapy
ARV: antiretroviral
CAB: cabotegravir
CDC: US Centers for Disease Control and Prevention
DDD: difference-in-difference-in-differences
DID: difference-in-differences
EHE: Ending the HIV Epidemic
FDA: Food and Drug Administration
FTC: fluoro-thiacytidine
HCPCS: Healthcare Common Procedure Coding System
ICD-9-CM: International Classification of Diseases, Ninth Revision, Clinical Modification
ICD-10-CM: International Classification of Diseases, Tenth Revision, Clinical Modification
IRB: institutional review board
LAI: long-acting injectable
LEM: Localized Economic Modeling
NASTAD: National Alliance of State and Territorial AIDS Directors
PDC: proportion of days covered
PEP: postexposure prophylaxis
PnR: PrEP-to-need ratio
PREMISE: PrEP Epidemiology, Modeling, and Surveillance
PrEP: pre-exposure prophylaxis
PrEP DAP: Pre-Exposure Prophylaxis Drug Assistance Program
RECORD: Reporting of Studies Conducted Using Observational Routinely Collected Data
TAF: tenofovir alafenamide fumarate
TDF: tenofovir disoproxil fumarate

## Data Availability

Proprietary prescription, diagnosis, and procedure claims data were purchased through a contract agreement with a third-party data provider. Data for this publication are not available for public sharing.
