# Supplementary material for: Establish a PrEP (Pre-Exposure Prophylaxis) Epidemiology, Modeling, and Surveillance (PREMISE) System to Analyze Trends in PrEP Uptake and the Impact of PrEP Programs and Policies: Protocol for a Natural Experiment and Modeling Study in the United States
Source: JMIR Res Protoc. 2026 Jan 30;15:e80911. doi: 10.2196/80911 (PMC12905565; doi:10.2196/80911)
Supplement: Multimedia Appendix 3 [file resprot_v15i1e80911_app3.docx]

**Multimedia Appendix 3: List of ICD-9-CM and ICD-10-CM codes related to HIV Conditions**

Table 1. Diagnoses codes that define HIV and opportunistic infections

| **Condition** | **Exclusion Items** | **ICD-9-CM** | **ICD-10-CM** |
| --- | --- | --- | --- |
| HIV | HIV disease | 042 | B20 |
|  | Asymptomatic HIV infection | V08 | Z21 |
|  | HIV-2 infection | 079.53 | B97.35 |
|  | HIV complicating pregnancy | NA | O98.7X* |
|  | Nonspecific serologic evidence of HIV | 795.71 | R75 |
| Opportunistic Infections | Candidiasis of bronchi, trachea, or esophagus | 112.84 | B37.81 |
|  | Candidiasis of lungs | 112.4 | B37.1 |
|  | Toxoplasmosis | 130.X* | B58.X* |
|  | Coccidioidomycosis | 114.X* | B38**.**X* |
|  | Cryptococcosis | 117.5 | B45**.**X* |
|  | Cryptosporidiosis | 007.4 | A07.2 |
|  | CMV retinitis | 078.5 | B25.8 |
|  | Kaposi’s sarcoma | 176.X | C46**.**X* |
|  | Mycobacterium avium complex | 031.2, 031.0 | A31.0, A31.2 |
|  | Pneumocystis carinii pneumonia | 136.3 | B59 |
| *‘X’ denotes any letter(s) or number after the decimal point. | | | |
