# Supplementary material for: Establish a PrEP (Pre-Exposure Prophylaxis) Epidemiology, Modeling, and Surveillance (PREMISE) System to Analyze Trends in PrEP Uptake and the Impact of PrEP Programs and Policies: Protocol for a Natural Experiment and Modeling Study in the United States
Source: JMIR Res Protoc. 2026 Jan 30;15:e80911. doi: 10.2196/80911 (PMC12905565; doi:10.2196/80911)
Supplement: Supplementary file 3 [file resprot_v15i1e80911_app2.doc]

# PrEP Impact Evaluation Survey 2025

---

## Introduction

 

**The Emory
University Rollins School of Public Health and NASTAD are collaborating to
conduct a survey of health departments to characterize and assess the impact of
PrEP support interventions in the US in terms of PrEP use and PrEP equity. This
survey is supported by a National Institutes of Health grant (1R01AI181799-01)
that is focused on analyzing PrEP programs and policies. Results will be
returned to health departments, and used to provide vital information to policy
makers, funders, and advocates.   
   
This survey has questions regarding eight types of PrEP promotion programs that
may have been conducted in your jurisdiction. Data will be kept confidential,
with only project researchers and staff having access to information provided.
Data will be stored securely in encrypted Emory databases.   
  
This survey autosaves once you mark an answer, so you can stop at any point and
not lose your entered answers. To continue, simply click on the secure link we
sent you to restart where you left off. The access link is unique to your
jurisdiction, which enables you to share it with other staff at your health
department without creating duplicate entries.**

 

---

## Contact Information

 

### 1) Individual completing the survey\*

First Name:
\_\_\_\_\_\_\_\_\_\_\_\_\_\_\_\_\_\_\_\_\_\_\_\_\_\_\_\_\_\_\_\_\_\_\_\_\_\_\_\_\_\_\_\_\_\_\_\_\_

Last Name:
\_\_\_\_\_\_\_\_\_\_\_\_\_\_\_\_\_\_\_\_\_\_\_\_\_\_\_\_\_\_\_\_\_\_\_\_\_\_\_\_\_\_\_\_\_\_\_\_\_

Title: \_\_\_\_\_\_\_\_\_\_\_\_\_\_\_\_\_\_\_\_\_\_\_\_\_\_\_\_\_\_\_\_\_\_\_\_\_\_\_\_\_\_\_\_\_\_\_\_\_

Organization Name:
\_\_\_\_\_\_\_\_\_\_\_\_\_\_\_\_\_\_\_\_\_\_\_\_\_\_\_\_\_\_\_\_\_\_\_\_\_\_\_\_\_\_\_\_\_\_\_\_\_

Email Address:
\_\_\_\_\_\_\_\_\_\_\_\_\_\_\_\_\_\_\_\_\_\_\_\_\_\_\_\_\_\_\_\_\_\_\_\_\_\_\_\_\_\_\_\_\_\_\_\_\_

Contact Number:
\_\_\_\_\_\_\_\_\_\_\_\_\_\_\_\_\_\_\_\_\_\_\_\_\_\_\_\_\_\_\_\_\_\_\_\_\_\_\_\_\_\_\_\_\_\_\_\_\_

 

#### 2) Do you work for the State or a County health department?\*

( ) State

( ) County

 

#### 3) Please select your State\*

( ) Alaska

( ) Arizona

( ) Arkansas

( ) Alabama

( ) California

( ) Colorado

( ) Connecticut

( ) Delaware

( ) Florida

( ) Georgia

( ) Hawaii

( ) Idaho

( ) Illinois

( ) Indiana

( ) Iowa

( ) Kansas

( ) Kentucky

( ) Louisiana

( ) Maine

( ) Maryland

( ) Massachusetts

( ) Michigan

( ) Minnesota

( ) Mississippi

( ) Missouri

( ) Montana

( ) Nebraska

( ) Nevada

( ) New Hampshire

( ) New Jersey

( ) New Mexico

( ) New York

( ) North Carolina

( ) North Dakota

( ) Ohio

( ) Oklahoma

( ) Oregon

( ) Pennsylvania

( ) Rhode Island

( ) South Carolina

( ) South Dakota

( ) Tennessee

( ) Texas

( ) Utah

( ) Vermont

( ) Virginia

( ) Washington

( ) Washington, D.C.

( ) West Virginia

( ) Wisconsin

( ) Wyoming

 

#### 4) Please indicate if any local EHE jurisdiction (s) are included in your response (check all that apply)\*

[ ] Maricopa County, Arizona

[ ] Alameda County, California

[ ] Los Angeles County, California

[ ] Orange County, California

[ ] Riverside County, California

[ ] Sacramento County, California

[ ] San Bernardino County,
California

[ ] San Diego County, California

[ ] San Francisco County,
California

[ ] Broward County, Florida

[ ] Duval County, Florida

[ ] Hillsborough County, Florida

[ ] Miami-Dade County, Florida

[ ] Orange County, Florida

[ ] Palm Beach County, Florida

[ ] Pinellas County, Florida

[ ] Cobb County, Georgia

[ ] DeKalb County, Georgia

[ ] Fulton County, Georgia

[ ] Gwinnett County, Georgia

[ ] Cook County, Illinois

[ ] Marion County, Indiana

[ ] East Baton Rouge Parish,
Louisiana

[ ] Orleans Parish, Louisiana

[ ] Baltimore City, Maryland

[ ] Montgomery County, Maryland

[ ] Prince George's County,
Maryland

[ ] Suffolk County, Massachusetts

[ ] Wayne County, Michigan

[ ] Clark County, Nevada

[ ] Essex County, New Jersey

[ ] Hudson County, New Jersey

[ ] Bronx County, New York

[ ] Kings County, New York

[ ] New York County, New York

[ ] Queens County, New York

[ ] Mecklenburg County, North
Carolina

[ ] Cuyahoga County, Ohio

[ ] Franklin County, Ohio

[ ] Hamilton County, Ohio

[ ] Philadelphia County,
Pennsylvania

[ ] Shelby County, Tennessee

[ ] Bexar County, Texas

[ ] Dallas County, Texas

[ ] Harris County, Texas

[ ] Tarrant County, Texas

[ ] Travis County, Texas

[ ] King County, Washington

[ ] San Juan, Puerto Rico

[ ] Washington, District of
Columbia

[ ] N/A

 

---

## Overview of Interventions

**For this
survey, we want to characterize eight different categories of PrEP promotion
programs. Understanding each of these and how they are different from each
other is critical for the study. We will provide definitions for each program
type as you progress through the survey.  Please briefly review these
categories to familiarize yourself with the eight categories. For
programs/initiatives/interventions that overlap, please include them in only
one category, based on the program’s primary aim.**

**1.****Messaging
Campaign** **are digital or
physical messaging, social marketing aimed at raising awareness about PrEP or
sharing information about PrEP services available**

**2.****PrEP
Outreach and Education programs****are non-clinical outreach and education activities, typically delivered by
staff or peers at community-based organizations (CBOs). They typically focus on
raising awareness about PrEP, addressing misinformation, and sharing
information about the availability of PrEP**

**3.****PrEP
Navigation** **services include
assistance to find a PrEP provider and addressing
barriers to access PrEP, as well as support strategies to facilitate retention
or re-initiation into PrEP care**

**4.****Telemedicine
PrEP** **consists of full or
hybrid telemedicine PrEP services that reduce or eliminate the need for
in-person clinical visits**

**5.****Safety-Net
PrEP Clinic** **refers to the
delivery of PrEP through an existing STI clinic, community health center,
pharmacy, or similar public health clinic, aimed at expanding PrEP access among
key populations through reduced cost or free services.**

**6.****PrEP
Assistance Programs** **are
programs that cover the cost of PrEP-related services, either directly or
indirectly usually by reimbursing providers for clinical visits and lab tests,
or paying PrEP-related out-of-pocket costs to health plans**

**7.****Electronic
Medical/Health Record (EMR/EHR) Optimization** **strategies include changes to eHRs to ease PrEP
delivery. This includes electronic reminders for providers to assess a patient
for PrEP eligibility, or developing a list of PrEP candidates using eHR
data**

**8.****Training
for Clinicians and Clinical Staff** **refer to training or organizational capacity building to increase PrEP
prescribing**

 

#### 5) Based on the categories and the examples provided, please indicate if your organization implemented any of the following programs between January 2016 and December 2024 (Check all that apply)\*

[ ] Messaging Campaign

[ ] PrEP Outreach and Education
Programs

[ ] PrEP Navigation

[ ] Telemedicine PrEP

[ ] Safety-Net PrEP Clinic

[ ] PrEP Assistance Programs

[ ] EMR/EHR Optimization

[ ] Training for Clinicians and
Clinical Staff

[ ] Other - Write In (Required):
\_\_\_\_\_\_\_\_\_\_\_\_\_\_\_\_\_\_\_\_\_\_\_\_\_\_\_\_\_\_\_\_\_\_\_\_\_\_\_\_\_\_\_\_\_\_\_\_\_\*

 

---

## Messaging Campaign

#### 6)

**Has your organization implemented **messaging
campaigns**?**

·       
**Include instances of digital or physical
messaging aimed to promote PrEP awareness and uptake**

·       
**Include social marketing, social media, print
media, partnership with radio station, public service announcement (PSA)**

·       
**Include activities conducted in-house or
through subcontracted partners**

#### \*

( ) Yes

( ) No

 

### 7) What is the name of the messaging campaign program in your jurisdiction? (please indicate "NA" if there is no specific name for the program)\*

\_\_\_\_\_\_\_\_\_\_\_\_\_\_\_\_\_\_\_\_\_\_\_\_\_\_\_\_\_\_\_\_\_\_\_\_\_\_\_\_\_\_\_\_\_\_\_\_\_

 

**8)
Approximately, when did your messaging campaign launch?**

**Federal Fiscal Year:** unknown, before
2016, 2016, 2017, 2018 …… 2025

**Quarter:** unknown, before 2016, 1, 2, 3,
4

**9) End date
of messaging campaign?**

**Federal Fiscal Year:** unknown, ongoing
2016, 2017, 2018 …… 2025

**Quarter:** unknown, ongoing, 1, 2, 3, 4

#### 

#### 10) What were the primary aims of the messaging campaign(s)?\*

[ ] Increase PrEP knowledge among
providers

[ ] Increase PrEP awareness among
potential PrEP candidates

[ ] Identify an underserved target
audience that could benefit from PrEP

[ ] Address misinformation about
PrEP and combat stigma

[ ] Facilitate access to PrEP by
connecting PrEP candidates to providers

[ ] Publicize a PrEP-related
resource/service

[ ] Other - Write In (Required):
\_\_\_\_\_\_\_\_\_\_\_\_\_\_\_\_\_\_\_\_\_\_\_\_\_\_\_\_\_\_\_\_\_\_\_\_\_\_\_\_\_\_\_\_\_\_\_\_\_\*

 

#### 11) Which media channels did the campaign(s) use?\*

[ ] Social media platforms,
including search engine marketing

[ ] Dating/hookup aps, including
ads and profiles

[ ] Print media, including
magazines, community publications, bus stop banners, and billboards

[ ] Place-based community media,
including posters in clinics and community venues

[ ] Television and radio,
including public service announcements

[ ] Other - Write In (Required):
\_\_\_\_\_\_\_\_\_\_\_\_\_\_\_\_\_\_\_\_\_\_\_\_\_\_\_\_\_\_\_\_\_\_\_\_\_\_\_\_\_\_\_\_\_\_\_\_\_\*

 

### For the following questions, you will be asked to indicate the funding source for the program and estimated total program costs expended.

#### 12) Funding source used for **messaging campaign** (check all that apply)\*

[ ] CDC PS12-1201 (2012-2018)

[ ] CDC PS18-1802 (2018-2024)

[ ] CDC PS20-2010 (2020-2024)

[ ] CDC PS24-0047 (2024-2029)

[ ] CDC PS-24-0047 PrEP
Supplemental (2024-2025)

[ ] Other CDC funding - Write In:
\_\_\_\_\_\_\_\_\_\_\_\_\_\_\_\_\_\_\_\_\_\_\_\_\_\_\_\_\_\_\_\_\_\_\_\_\_\_\_\_\_\_\_\_\_\_\_\_\_

[ ] Other Federal funding - Write
In: \_\_\_\_\_\_\_\_\_\_\_\_\_\_\_\_\_\_\_\_\_\_\_\_\_\_\_\_\_\_\_\_\_\_\_\_\_\_\_\_\_\_\_\_\_\_\_\_\_

[ ] State appropriation

[ ] Other State funding - Write
In: \_\_\_\_\_\_\_\_\_\_\_\_\_\_\_\_\_\_\_\_\_\_\_\_\_\_\_\_\_\_\_\_\_\_\_\_\_\_\_\_\_\_\_\_\_\_\_\_\_

[ ] Subrecipient of state funding

[ ] Subrecipient of federal
funding

[ ] Local funding

[ ] Private funding

[ ] 340B revenue / program income

[ ] Other - Write In:
\_\_\_\_\_\_\_\_\_\_\_\_\_\_\_\_\_\_\_\_\_\_\_\_\_\_\_\_\_\_\_\_\_\_\_\_\_\_\_\_\_\_\_\_\_\_\_\_\_

#### Comments:

 

 

---

## Messaging Campaign

### 13) [For each of the funding streaming selected above]

**Please provide
the estimated total cost/budget expended on this intervention. If actual
expenditures are not available, budgets may be used to estimate costs. You will
have the options to provide the dollar value of the estimated cost/budget or
provide the percentage of cost/budget, or both.**

**If providing** dollar value, please round it
to the nearest $50,000. If less than $25,000, please mark as $0.:
\_\_\_\_\_\_\_\_\_\_\_\_\_\_\_\_\_\_\_\_\_\_\_\_\_\_\_\_\_\_\_\_\_\_\_\_\_\_\_\_\_\_\_\_\_\_\_\_\_

**If providing** percentages, it should be
calculated across all selected programs. The percentages should add up to 100%
across all programs and funding streams.:
\_\_\_\_\_\_\_\_\_\_\_\_\_\_\_\_\_\_\_\_\_\_\_\_\_\_\_\_\_\_\_\_\_\_\_\_\_\_\_\_\_\_\_\_\_\_\_\_\_

#### Comments:

 

 

#### [For each of the funding streaming selected above]

#### 14) Please indicate for [the period input for launch and end date], the funding covered:

( ) All of these years

( ) Some of these years

#### Comments:

 

 

---

## PrEP Outreach and Education

#### 15)

**Has your organization implemented a **PrEP** **outreach and education program**?**

·       
**Include programs aimed at increasing PrEP
awareness in key populations and PrEP promotion activities**

·       
**Include CBO-led outreach, education, or any
other CBO-led initiatives to promote PrEP awareness and population-level
uptake**

·       
**Include activities carried out in-house or
through subcontracted partners**

·       
**DO NOT include programs primarily dedicated
to linking individuals to PrEP services, which will be covered next**

#### \*

( ) Yes

( ) No

**16)
Approximately, when did your PrEP outreach and
education program launch?**

**Federal Fiscal Year:** unknown, before
2016, 2016, 2017, 2018 …… 2025

**Quarter:** unknown, before 2016, 1, 2, 3,
4

**17) End date
of PrEP outreach and education program?**

**Federal Fiscal Year:** unknown, ongoing
2016, 2017, 2018 …… 2025

**Quarter:** unknown, ongoing, 1, 2, 3, 4

 

#### 18) Did your PrEP outreach and education initiatives include any of the following components?\*

[ ] Distribution of supplies:
condoms and lube, sterile syringes and injection equipment, naloxone, or wound
care supplies

[ ] HIV/STI/hepatitis C screening
in connection with PrEP education

[ ] Distribution of educational
materials

[ ] Risk reduction counseling that
includes information about PrEP

[ ] Other - Write In (Required):
\_\_\_\_\_\_\_\_\_\_\_\_\_\_\_\_\_\_\_\_\_\_\_\_\_\_\_\_\_\_\_\_\_\_\_\_\_\_\_\_\_\_\_\_\_\_\_\_\_\*

 

### For the following questions, you will be asked to indicate the funding source for the program and estimated total program costs expended.

#### 19) Funding source used for **PrEP outreach and education program** (check all that apply)\*

[ ] CDC PS12-1201 (2012-2018)

[ ] CDC PS18-1802 (2018-2024)

[ ] CDC PS20-2010 (2020-2024)

[ ] CDC PS24-0047 (2024-2029)

[ ] CDC PS-24-0047 PrEP
Supplemental (2024-2025)

[ ] Other CDC funding - Write In:
\_\_\_\_\_\_\_\_\_\_\_\_\_\_\_\_\_\_\_\_\_\_\_\_\_\_\_\_\_\_\_\_\_\_\_\_\_\_\_\_\_\_\_\_\_\_\_\_\_

[ ] Other Federal funding - Write
In: \_\_\_\_\_\_\_\_\_\_\_\_\_\_\_\_\_\_\_\_\_\_\_\_\_\_\_\_\_\_\_\_\_\_\_\_\_\_\_\_\_\_\_\_\_\_\_\_\_

[ ] State appropriation

[ ] Other State funding - Write
In: \_\_\_\_\_\_\_\_\_\_\_\_\_\_\_\_\_\_\_\_\_\_\_\_\_\_\_\_\_\_\_\_\_\_\_\_\_\_\_\_\_\_\_\_\_\_\_\_\_

[ ] Subrecipient of state funding

[ ] Subrecipient of federal
funding

[ ] Local funding

[ ] Private funding

[ ] 340B revenue / program income

[ ] Other - Write In:
\_\_\_\_\_\_\_\_\_\_\_\_\_\_\_\_\_\_\_\_\_\_\_\_\_\_\_\_\_\_\_\_\_\_\_\_\_\_\_\_\_\_\_\_\_\_\_\_\_

#### Comments:

 

 

---

## PrEP Outreach and Education

### 20) [For each of the funding streaming selected above]

**Please provide
the estimated total cost/budget expended on this intervention. If actual
expenditures are not available, budgets may be used to estimate costs. You will
have the options to provide the dollar value of the estimated cost/budget or
provide the percentage of cost/budget, or both.**

**If providing** dollar value, please round it
to the nearest $50,000. If less than $25,000, please mark as $0.:
\_\_\_\_\_\_\_\_\_\_\_\_\_\_\_\_\_\_\_\_\_\_\_\_\_\_\_\_\_\_\_\_\_\_\_\_\_\_\_\_\_\_\_\_\_\_\_\_\_

**If providing** percentages, it should be
calculated across all selected programs. The percentages should add up to 100%
across all programs and funding streams.:
\_\_\_\_\_\_\_\_\_\_\_\_\_\_\_\_\_\_\_\_\_\_\_\_\_\_\_\_\_\_\_\_\_\_\_\_\_\_\_\_\_\_\_\_\_\_\_\_\_

#### Comments:

 

### [For each of the funding streaming selected above]

#### 21) Please indicate for [the period input for launch and end date], the funding covered:

( ) All of these years

( ) Some of these years

#### Comments:

 

 

---

## PrEP Navigation

#### 22)

**Has your organization implemented a **PrEP** **navigation** program?**

·       
**Include PrEP navigation services such as
linkage to a PrEP provider, assistance with
insurance/benefits navigation, and behavioral counselling to determine PrEP
candidacy or to support PrEP adherence such as reminders to pick up
medications**

·       
**Include components of an HIV or STI testing
program dedicated to PrEP linkage, but DO NOT include the entire HIV or STI
testing program if the primary purpose of the program is not PrEP linkage (e,g. a PrEP linkage coordinator embedded in a targeted HIV
testing program)**

·       
**For status-neutral navigation and linkage,
estimate an allocation of resources dedicated to the PrEP components**

·       
**Include interventions aimed at facilitating
the retention of PrEP-eligible persons in PrEP care, including case management,
adherence counselling, and patient incentives**

·       
**Include programs delivered directly or
through subcontracted partners**

·       
**Include interventions and tools that leverage
technology of remove structural barriers such as mobile applications designed
to provide adherence support, a resource bank, or rideshare credits used to
address challenges getting to clinical appointments**

#### \*

( ) Yes

( ) No

**23)
Approximately, when did your PrEP Navigation program launch?**

**Federal Fiscal Year:** unknown, before
2016, 2016, 2017, 2018 …… 2025

**Quarter:** unknown, before 2016, 1, 2, 3,
4

**24) End date
of PrEP Navigation program?**

**Federal Fiscal Year:** unknown, ongoing
2016, 2017, 2018 …… 2025

**Quarter:** unknown, ongoing, 1, 2, 3, 4

 

#### 25) Did your PrEP navigation program(s) provide any of the following services?\*

[ ] HIV risk counseling to
determine PrEP candidacy

[ ] Linkage to a PrEP provider

[ ] Assistance with
insurance/benefits navigation

[ ] Behavioral counseling to
support PrEP adherence

[ ] Ongoing reminders about
appointments or medication pick-up

[ ] Ongoing case management
services

[ ] Text messaging and mobile
application communication

[ ] A resource bank

[ ] Clinical transportation
assistance

[ ] Patient incentives

[ ] Status-neutral navigation
services

[ ] Other - Write In (Required):
\_\_\_\_\_\_\_\_\_\_\_\_\_\_\_\_\_\_\_\_\_\_\_\_\_\_\_\_\_\_\_\_\_\_\_\_\_\_\_\_\_\_\_\_\_\_\_\_\_\*

 

### For the following questions, you will be asked to indicate the funding source for the program and estimated total program costs expended.

#### 26) Funding source used for **PrEP Navigation program** (check all that apply)\*

[ ] CDC PS12-1201 (2012-2018)

[ ] CDC PS18-1802 (2018-2024)

[ ] CDC PS20-2010 (2020-2024)

[ ] CDC PS24-0047 (2024-2029)

[ ] CDC PS-24-0047 PrEP
Supplemental (2024-2025)

[ ] Other CDC funding - Write In:
\_\_\_\_\_\_\_\_\_\_\_\_\_\_\_\_\_\_\_\_\_\_\_\_\_\_\_\_\_\_\_\_\_\_\_\_\_\_\_\_\_\_\_\_\_\_\_\_\_

[ ] Other Federal funding - Write
In: \_\_\_\_\_\_\_\_\_\_\_\_\_\_\_\_\_\_\_\_\_\_\_\_\_\_\_\_\_\_\_\_\_\_\_\_\_\_\_\_\_\_\_\_\_\_\_\_\_

[ ] State appropriation

[ ] Other State funding - Write
In: \_\_\_\_\_\_\_\_\_\_\_\_\_\_\_\_\_\_\_\_\_\_\_\_\_\_\_\_\_\_\_\_\_\_\_\_\_\_\_\_\_\_\_\_\_\_\_\_\_

[ ] Subrecipient of state funding

[ ] Subrecipient of federal
funding

[ ] Local funding

[ ] Private funding

[ ] 340B revenue / program income

[ ] Other - Write In:
\_\_\_\_\_\_\_\_\_\_\_\_\_\_\_\_\_\_\_\_\_\_\_\_\_\_\_\_\_\_\_\_\_\_\_\_\_\_\_\_\_\_\_\_\_\_\_\_\_

#### Comments:

 

 

---

## PrEP Navigation

 

### 27) [For each of the funding streaming selected above]

**Please provide
the estimated total cost/budget expended on this intervention. If actual
expenditures are not available, budgets may be used to estimate costs. You will
have the options to provide the dollar value of the estimated cost/budget or
provide the percentage of cost/budget, or both.**

**If providing** dollar value, please round it
to the nearest $50,000. If less than $25,000, please mark as $0.:
\_\_\_\_\_\_\_\_\_\_\_\_\_\_\_\_\_\_\_\_\_\_\_\_\_\_\_\_\_\_\_\_\_\_\_\_\_\_\_\_\_\_\_\_\_\_\_\_\_

**If providing** percentages, it should be
calculated across all selected programs. The percentages should add up to 100%
across all programs and funding streams.:
\_\_\_\_\_\_\_\_\_\_\_\_\_\_\_\_\_\_\_\_\_\_\_\_\_\_\_\_\_\_\_\_\_\_\_\_\_\_\_\_\_\_\_\_\_\_\_\_\_

#### Comments:

 

**[For each of the funding streaming selected above]**

#### 28) Please indicate for [the period input for launch and end date], the funding covered:

( ) All of these years

( ) Some of these years

#### Comments:

 

 

---

## Telemedicine PrEP

#### 29)

**Has your organization implemented a **telemedicine/remote
PrEP program**?**

·       
**Include programs that provide fully virtual
or hybrid telemedicine PrEP services (e.g. remote visits and in-person
labs)**

·       
**Include telemedicine models with in-person
visit requirements if the majority of visits are
delivered virtually**

·       
**Include telemedicine PrEP services delivered
directly or through subcontracted partners**

#### \*

( ) Yes

( ) No

**30)
Approximately, when did your telemedicine/remote PrEP program launch?**

**Federal Fiscal Year:** unknown, before
2016, 2016, 2017, 2018 …… 2025

**Quarter:** unknown, before 2016, 1, 2, 3,
4

**31) End date
of telemedicine/remote PrEP program?**

**Federal Fiscal Year:** unknown, ongoing
2016, 2017, 2018 …… 2025

**Quarter:** unknown, ongoing, 1, 2, 3, 4

 

#### 32) Did your PrEP telemedicine program(s) provide any of the following services?\*

[ ] Patient portal or messaging
system for patients to communicate with the provider/team

[ ] Initial assessment to initiate
PrEP conducted virtually

[ ] Virtual visits for PrEP
follow-up/management

[ ] STI treatment protocols for
managing positive STI results, which may include e-prescribing appropriate
antibiotics or referring the patient to a local provider if an injection or
in-person evaluation is necessary

[ ] Remote lab test orders
provided to the patient

[ ] Lab testing through local
partnerships allowing patients to visit national chains or local
hospital/clinic labs

[ ] At-home self-collection kits
mailed directly to patients for self-collection of specimens (finger-prick for
dried blood spot tests, urine samples, and rectal/vaginal/throat swabs)

[ ] Electronic prescriptions sent
to the patient's chosen local pharmacy

[ ] Mail-order pharmacy that ships
PrEP drugs to the patient's home

[ ] Onboarding assistance to set
up accounts, scheduling virtual appointments, or coordinating lab testing
logistics

[ ] Benefits navigation, including
insurance coverage, enrollment in Patient Assistance Programs, or co-pay
assistance programs

[ ] Adherence support, including
reminders, tools, and ongoing counseling to support consistent medication use

[ ] Other - Write In (Required):
\_\_\_\_\_\_\_\_\_\_\_\_\_\_\_\_\_\_\_\_\_\_\_\_\_\_\_\_\_\_\_\_\_\_\_\_\_\_\_\_\_\_\_\_\_\_\_\_\_\*

 

### For the following questions, you will be asked to indicate the funding source for the program and estimated total program costs expended.

#### 33) Funding source used for **telemedicine/remote PrEP program** (check all that apply)\*

[ ] CDC PS12-1201 (2012-2018)

[ ] CDC PS18-1802 (2018-2024)

[ ] CDC PS20-2010 (2020-2024)

[ ] CDC PS24-0047 (2024-2029)

[ ] CDC PS-24-0047 PrEP
Supplemental (2024-2025)

[ ] Other CDC funding - Write In:
\_\_\_\_\_\_\_\_\_\_\_\_\_\_\_\_\_\_\_\_\_\_\_\_\_\_\_\_\_\_\_\_\_\_\_\_\_\_\_\_\_\_\_\_\_\_\_\_\_

[ ] Other Federal funding - Write
In: \_\_\_\_\_\_\_\_\_\_\_\_\_\_\_\_\_\_\_\_\_\_\_\_\_\_\_\_\_\_\_\_\_\_\_\_\_\_\_\_\_\_\_\_\_\_\_\_\_

[ ] State appropriation

[ ] Other State funding - Write
In: \_\_\_\_\_\_\_\_\_\_\_\_\_\_\_\_\_\_\_\_\_\_\_\_\_\_\_\_\_\_\_\_\_\_\_\_\_\_\_\_\_\_\_\_\_\_\_\_\_

[ ] Subrecipient of state funding

[ ] Subrecipient of federal
funding

[ ] Local funding

[ ] Private funding

[ ] 340B revenue / program income

[ ] Other - Write In:
\_\_\_\_\_\_\_\_\_\_\_\_\_\_\_\_\_\_\_\_\_\_\_\_\_\_\_\_\_\_\_\_\_\_\_\_\_\_\_\_\_\_\_\_\_\_\_\_\_

#### Comments:

 

 

---

## Telemedicine PrEP

### 34) [For each of the funding streaming selected above]

**Please provide
the estimated total cost/budget expended on this intervention. If actual
expenditures are not available, budgets may be used to estimate costs. You will
have the options to provide the dollar value of the estimated cost/budget or
provide the percentage of cost/budget, or both.**

**If providing** dollar value, please round it
to the nearest $50,000. If less than $25,000, please mark as $0.:
\_\_\_\_\_\_\_\_\_\_\_\_\_\_\_\_\_\_\_\_\_\_\_\_\_\_\_\_\_\_\_\_\_\_\_\_\_\_\_\_\_\_\_\_\_\_\_\_\_

**If providing** percentages, it should be
calculated across all selected programs. The percentages should add up to 100%
across all programs and funding streams.:
\_\_\_\_\_\_\_\_\_\_\_\_\_\_\_\_\_\_\_\_\_\_\_\_\_\_\_\_\_\_\_\_\_\_\_\_\_\_\_\_\_\_\_\_\_\_\_\_\_

#### Comments:

 

 

 

 

**[For each of the funding streaming selected above]**

#### 35) Please indicate for [the period input for launch and end date], the funding covered:

( ) All of these years

( ) Some of these years

#### Comments:

 

 

---

## Safety-Net PrEP Clinic

#### 36)

**Has your organization implemented a **Safety-Net
PrEP clinic**?**

·       
**Include PrEP delivery through new or existing
state or local health department clinics, such as STI clinics, through family
planning clinics, community health centers (Federally Qualified Health Centers
and look-alikes), pharmacies, or similar safety-net healthcare programs that
seek to increase access to PrEP prescribing**

·       
**Include clinics operated by the health
department or through subcontracted partners**

·       
**Include staffing cost related to the
operation of the PrEP clinic**

·       
**DO NOT include telemedicine PrEP delivery or
private providers that receive funds through a PrEP Assistance Program.**

#### \*

( ) Yes

( ) No

**37) Approximately, when did your **Safety-Net
PrEP clinic** program launch?**

**Federal Fiscal Year:** unknown, before
2016, 2016, 2017, 2018 …… 2025

**Quarter:** unknown, before 2016, 1, 2, 3,
4

**38) End date of **Safety-Net PrEP
clinic**?**

**Federal Fiscal Year:** unknown, ongoing
2016, 2017, 2018 …… 2025

**Quarter:** unknown, ongoing, 1, 2, 3, 4

 

#### 39) Which types of Safety-Net PrEP clinics have you funded? (Check all that apply)\*

[ ] State health department clinic

[ ] Local health department STI
clinic

[ ] Family Planning clinic

[ ] Community health center

[ ] AIDS Service Organization/CBO
offering a PrEP clinic

[ ] Local pharmacy

[ ] Commercial pharmacy

[ ] Other - Write In (Required):
\_\_\_\_\_\_\_\_\_\_\_\_\_\_\_\_\_\_\_\_\_\_\_\_\_\_\_\_\_\_\_\_\_\_\_\_\_\_\_\_\_\_\_\_\_\_\_\_\_\*

 

### For the following questions, you will be asked to indicate the funding source for the program and estimated total program costs expended.

#### 40) Funding source used for Safety-Net PrEP clinic (check all that apply)\*

[ ] CDC PS12-1201 (2012-2018)

[ ] CDC PS18-1802 (2018-2024)

[ ] CDC PS20-2010 (2020-2024)

[ ] CDC PS24-0047 (2024-2029)

[ ] CDC PS-24-0047 PrEP
Supplemental (2024-2025)

[ ] Other CDC funding - Write In:
\_\_\_\_\_\_\_\_\_\_\_\_\_\_\_\_\_\_\_\_\_\_\_\_\_\_\_\_\_\_\_\_\_\_\_\_\_\_\_\_\_\_\_\_\_\_\_\_\_

[ ] Other Federal funding - Write
In: \_\_\_\_\_\_\_\_\_\_\_\_\_\_\_\_\_\_\_\_\_\_\_\_\_\_\_\_\_\_\_\_\_\_\_\_\_\_\_\_\_\_\_\_\_\_\_\_\_

[ ] State appropriation

[ ] Other State funding - Write
In: \_\_\_\_\_\_\_\_\_\_\_\_\_\_\_\_\_\_\_\_\_\_\_\_\_\_\_\_\_\_\_\_\_\_\_\_\_\_\_\_\_\_\_\_\_\_\_\_\_

[ ] Subrecipient of state funding

[ ] Subrecipient of federal
funding

[ ] Local funding

[ ] Private funding

[ ] 340B revenue / program income

[ ] Other - Write In:
\_\_\_\_\_\_\_\_\_\_\_\_\_\_\_\_\_\_\_\_\_\_\_\_\_\_\_\_\_\_\_\_\_\_\_\_\_\_\_\_\_\_\_\_\_\_\_\_\_

#### Comments:

 

 

---

## Safety-Net PrEP Clinic

**41)** **[For each of
the funding streaming selected above]**

**Please provide
the estimated total cost/budget expended on this intervention. If actual
expenditures are not available, budgets may be used to estimate costs. You will
have the options to provide the dollar value of the estimated cost/budget or
provide the percentage of cost/budget, or both.**

**If providing** dollar value, please round it
to the nearest $50,000. If less than $25,000, please mark as $0.:
\_\_\_\_\_\_\_\_\_\_\_\_\_\_\_\_\_\_\_\_\_\_\_\_\_\_\_\_\_\_\_\_\_\_\_\_\_\_\_\_\_\_\_\_\_\_\_\_\_

**If providing** percentages, it should be
calculated across all selected programs. The percentages should add up to 100%
across all programs and funding streams.:
\_\_\_\_\_\_\_\_\_\_\_\_\_\_\_\_\_\_\_\_\_\_\_\_\_\_\_\_\_\_\_\_\_\_\_\_\_\_\_\_\_\_\_\_\_\_\_\_\_

#### Comments:

 

**[For each of the funding streaming selected above]**

#### 42) Please indicate for [the period input for launch and end date], the funding covered:

( ) All of these years

( ) Some of these years

#### Comments:

 

 

---

## PrEP Assistance Program

#### 43)

**Has your organization implemented a **PrEP** **assistance program**?**

·       
**Include the use of any governmental funds,
including EHE funds, federal funds, State funds, local funds, or 340B
savings/program income**

·       
**Include programs that cover the cost of one
or more PrEP-related services, even if the program doesn’t cover all
PrEP-related services**

·       
**Include programs that fill gaps in existing
coverage, even if the program doesn’t cover the full cost of PrEP services, or
serves as payer-of-last-resort**

·       
**Include programs operated in-house or through
subcontracted partners**

#### \*

( ) Yes

( ) No

 

**44) Approximately, when did your **PrEP
Assistance** program launch?**

**Federal Fiscal Year:** unknown, before
2016, 2016, 2017, 2018 …… 2025

**Quarter:** unknown, before 2016, 1, 2, 3,
4

**45) End date of **PrEP Assistance
program**?**

**Federal Fiscal Year:** unknown, ongoing
2016, 2017, 2018 …… 2025

**Quarter:** unknown, ongoing, 1, 2, 3, 4

 

 

 

#### 46) What PrEP services does your PrEP assistance program cover? (Check all that apply)\*

[ ] PrEP clinical visit fees

[ ] PrEP-related lab tests

[ ] PrEP medication

[ ] Other PrEP-related services -
Write In (Required): \_\_\_\_\_\_\_\_\_\_\_\_\_\_\_\_\_\_\_\_\_\_\_\_\_\_\_\_\_\_\_\_\_\_\_\_\_\_\_\_\_\_\_\_\_\_\_\_\_\*

 

#### 47) How does your PrEP assistance program covers these costs? (Check all that apply)\*

[ ] Direct payment to
providers/laboratories

[ ] Direct medication purchase

[ ] Covering PrEP-related client
out-of-pocket costs

[ ] Covering health insurance
premium-related costs

[ ] Other - Write In (Required):
\_\_\_\_\_\_\_\_\_\_\_\_\_\_\_\_\_\_\_\_\_\_\_\_\_\_\_\_\_\_\_\_\_\_\_\_\_\_\_\_\_\_\_\_\_\_\_\_\_\*

 

### For the following questions, you will be asked to indicate the funding source for the program and estimated total program costs expended.

#### 48) Funding source used for **PrEP assistance program** (check all that apply)\*

[ ] CDC PS12-1201 (2012-2018)

[ ] CDC PS18-1802 (2018-2024)

[ ] CDC PS20-2010 (2020-2024)

[ ] CDC PS24-0047 (2024-2029)

[ ] CDC PS-24-0047 PrEP
Supplemental (2024-2025)

[ ] Other CDC funding - Write In:
\_\_\_\_\_\_\_\_\_\_\_\_\_\_\_\_\_\_\_\_\_\_\_\_\_\_\_\_\_\_\_\_\_\_\_\_\_\_\_\_\_\_\_\_\_\_\_\_\_

[ ] Other Federal funding - Write
In: \_\_\_\_\_\_\_\_\_\_\_\_\_\_\_\_\_\_\_\_\_\_\_\_\_\_\_\_\_\_\_\_\_\_\_\_\_\_\_\_\_\_\_\_\_\_\_\_\_

[ ] State appropriation

[ ] Other State funding - Write
In: \_\_\_\_\_\_\_\_\_\_\_\_\_\_\_\_\_\_\_\_\_\_\_\_\_\_\_\_\_\_\_\_\_\_\_\_\_\_\_\_\_\_\_\_\_\_\_\_\_

[ ] Subrecipient of state funding

[ ] Subrecipient of federal
funding

[ ] Local funding

[ ] Private funding

[ ] 340B revenue / program income

[ ] Other - Write In:
\_\_\_\_\_\_\_\_\_\_\_\_\_\_\_\_\_\_\_\_\_\_\_\_\_\_\_\_\_\_\_\_\_\_\_\_\_\_\_\_\_\_\_\_\_\_\_\_\_

#### Comments:

 

 

---

## PrEP Assistance Program

**49)** **[For each of
the funding streaming selected above]**

**Please provide
the estimated total cost/budget expended on this intervention. If actual
expenditures are not available, budgets may be used to estimate costs. You will
have the options to provide the dollar value of the estimated cost/budget or
provide the percentage of cost/budget, or both.**

**If providing** dollar value, please round it
to the nearest $50,000. If less than $25,000, please mark as $0.:
\_\_\_\_\_\_\_\_\_\_\_\_\_\_\_\_\_\_\_\_\_\_\_\_\_\_\_\_\_\_\_\_\_\_\_\_\_\_\_\_\_\_\_\_\_\_\_\_\_

**If providing** percentages, it should be
calculated across all selected programs. The percentages should add up to 100%
across all programs and funding streams.:
\_\_\_\_\_\_\_\_\_\_\_\_\_\_\_\_\_\_\_\_\_\_\_\_\_\_\_\_\_\_\_\_\_\_\_\_\_\_\_\_\_\_\_\_\_\_\_\_\_

#### Comments:

 

**[For each of the funding streaming selected above]**

#### 50) Please indicate for [the period input for launch and end date], the funding covered:

( ) All of these years

( ) Some of these years

#### Comments:

 

 

---

## EMR/EHR Optimizations

#### 51)

**Has your organization implemented **EMR/EHR
optimization programs**?**

·       
**Include programs related to the use or
improvement of EMR/EHR/related systems to support workflow processes related to
PrEP prescribing such as prompts for sexual health screening, PrEP eligibility,
or PrEP initiation**

·       
**Include activities conducted on internal
systems or for the systems used by clinical partners in the jurisdiction**

#### \*

( ) Yes

( ) No

**52) Approximately, when did your **EMR/EHR
optimization** program launch?**

**Federal Fiscal Year:** unknown, before
2016, 2016, 2017, 2018 …… 2025

**Quarter:** unknown, before 2016, 1, 2, 3,
4

**53) End date of **EMR/EHR optimization
program**?**

**Federal Fiscal Year:** unknown, ongoing
2016, 2017, 2018 …… 2025

**Quarter:** unknown, ongoing, 1, 2, 3, 4

 

#### 54) Did your EHR optimization initiatives include any of the following components?

[ ] Clinical decision support
(CDS) tools, including eligibility alerts, integrated HIV risk assessment
questions, contraindication warnings, or prompts for action based on guideline
recommendations

[ ] Workflow efficiency tools such
as PrEP-specific order sets, templated provider notes and documentation, PrEP
flowsheets, or e-prescribing capabilities

[ ] Patient engagement tools that
enable patients to schedule appointments, message with their care team, or
deliver educational materials and automated reminders

[ ] Population-level reporting and
quality improvement tools that track PrEP patient outcome rates, including PrEP
patient dashboards, as well as data extraction for outreach

[ ] Other - Write In (Required):
\_\_\_\_\_\_\_\_\_\_\_\_\_\_\_\_\_\_\_\_\_\_\_\_\_\_\_\_\_\_\_\_\_\_\_\_\_\_\_\_\_\_\_\_\_\_\_\_\_\*

 

### For the following questions, you will be asked to indicate the funding source for the program and estimated total program costs expended.

#### 55) Funding source used for **EMR/EHR optimization program** (check all that apply)\*

[ ] CDC PS12-1201 (2012-2018)

[ ] CDC PS18-1802 (2018-2024)

[ ] CDC PS20-2010 (2020-2024)

[ ] CDC PS24-0047 (2024-2029)

[ ] CDC PS-24-0047 PrEP
Supplemental (2024-2025)

[ ] Other CDC funding - Write In:
\_\_\_\_\_\_\_\_\_\_\_\_\_\_\_\_\_\_\_\_\_\_\_\_\_\_\_\_\_\_\_\_\_\_\_\_\_\_\_\_\_\_\_\_\_\_\_\_\_

[ ] Other Federal funding - Write
In: \_\_\_\_\_\_\_\_\_\_\_\_\_\_\_\_\_\_\_\_\_\_\_\_\_\_\_\_\_\_\_\_\_\_\_\_\_\_\_\_\_\_\_\_\_\_\_\_\_

[ ] State appropriation

[ ] Other State funding - Write
In: \_\_\_\_\_\_\_\_\_\_\_\_\_\_\_\_\_\_\_\_\_\_\_\_\_\_\_\_\_\_\_\_\_\_\_\_\_\_\_\_\_\_\_\_\_\_\_\_\_

[ ] Subrecipient of state funding

[ ] Subrecipient of federal
funding

[ ] Local funding

[ ] Private funding

[ ] 340B revenue / program income

[ ] Other - Write In:
\_\_\_\_\_\_\_\_\_\_\_\_\_\_\_\_\_\_\_\_\_\_\_\_\_\_\_\_\_\_\_\_\_\_\_\_\_\_\_\_\_\_\_\_\_\_\_\_\_

#### Comments:

 

 

---

## EMR/EHR Optimizations

### 56) [For each of the funding streaming selected above]

**Please provide
the estimated total cost/budget expended on this intervention. If actual
expenditures are not available, budgets may be used to estimate costs. You will
have the options to provide the dollar value of the estimated cost/budget or
provide the percentage of cost/budget, or both.**

**If providing** dollar value, please round it
to the nearest $50,000. If less than $25,000, please mark as $0.:
\_\_\_\_\_\_\_\_\_\_\_\_\_\_\_\_\_\_\_\_\_\_\_\_\_\_\_\_\_\_\_\_\_\_\_\_\_\_\_\_\_\_\_\_\_\_\_\_\_

**If providing** percentages, it should be
calculated across all selected programs. The percentages should add up to 100%
across all programs and funding streams.:
\_\_\_\_\_\_\_\_\_\_\_\_\_\_\_\_\_\_\_\_\_\_\_\_\_\_\_\_\_\_\_\_\_\_\_\_\_\_\_\_\_\_\_\_\_\_\_\_\_

#### Comments:

 

**[For each of the funding streaming selected above]**

#### 57) Please indicate for [the period input for launch and end date], the funding covered:

( ) All of these years

( ) Some of these years

#### Comments:

 

 

---

## Training for Clinicians and Clinical Staff

#### 58)

**Has your organization delivered **training
or organizational capacity-building assistance (CBA) programs for clinicians
and clinical staff**?**

·       
**Include training and CBA for clinicians,
clinical staff, clinical management teams, or clinic outreach staff, whether
delivered to funded or unfunded providers**

·       
**Include training and CBA activities delivered
directly or through subcontracted partners**

·       
**DO NOT include changes to EMR, clinic data
systems, clinic navigation programs, telemedicine PrEP, or PrEP clinic
implementation**

#### \*

( ) Yes

( ) No

**59) Approximately, when did your training
for clinicians and clinical staff program launch?**

**Federal Fiscal Year:** unknown, before
2016, 2016, 2017, 2018 …… 2025

**Quarter:** unknown, before 2016, 1, 2, 3,
4

**60) End date of training for clinicians and
clinical staff **program**?**

**Federal Fiscal Year:** unknown, ongoing
2016, 2017, 2018 …… 2025

**Quarter:** unknown, ongoing, 1, 2, 3, 4

 

#### 61) Did your training/TA efforts include any of the following components?\*

[ ] Training to improve clinical
knowledge about PrEP, including clinical guidelines and addressing provider
concerns

[ ] Cultural competency and bias
reduction training

[ ] Counselling skills training to
improve sexual health discussions

[ ] Implementation TA to develop
standard PrEP protocol development or integrate workflows

[ ] Resource distribution,
including provider checklists or patient education materials

[ ] Practice transformation TA
that includes team-based training and external coaching to facilitate
clinic-wide implementation

[ ] Other - Write In (Required):
\_\_\_\_\_\_\_\_\_\_\_\_\_\_\_\_\_\_\_\_\_\_\_\_\_\_\_\_\_\_\_\_\_\_\_\_\_\_\_\_\_\_\_\_\_\_\_\_\_\*

 

### For the following questions, you will be asked to indicate the funding source for the program and estimated total program costs expended.

#### 62) Funding source used for training for **clinicians and clinical staff program** (check all that apply)\*

[ ] CDC PS12-1201 (2012-2018)

[ ] CDC PS18-1802 (2018-2024)

[ ] CDC PS20-2010 (2020-2024)

[ ] CDC PS24-0047 (2024-2029)

[ ] CDC PS-24-0047 PrEP
Supplemental (2024-2025)

[ ] Other CDC funding - Write In:
\_\_\_\_\_\_\_\_\_\_\_\_\_\_\_\_\_\_\_\_\_\_\_\_\_\_\_\_\_\_\_\_\_\_\_\_\_\_\_\_\_\_\_\_\_\_\_\_\_

[ ] Other Federal funding - Write
In: \_\_\_\_\_\_\_\_\_\_\_\_\_\_\_\_\_\_\_\_\_\_\_\_\_\_\_\_\_\_\_\_\_\_\_\_\_\_\_\_\_\_\_\_\_\_\_\_\_

[ ] State appropriation

[ ] Other State funding - Write
In: \_\_\_\_\_\_\_\_\_\_\_\_\_\_\_\_\_\_\_\_\_\_\_\_\_\_\_\_\_\_\_\_\_\_\_\_\_\_\_\_\_\_\_\_\_\_\_\_\_

[ ] Subrecipient of state funding

[ ] Subrecipient of federal
funding

[ ] Local funding

[ ] Private funding

[ ] 340B revenue / program income

[ ] Other - Write In:
\_\_\_\_\_\_\_\_\_\_\_\_\_\_\_\_\_\_\_\_\_\_\_\_\_\_\_\_\_\_\_\_\_\_\_\_\_\_\_\_\_\_\_\_\_\_\_\_\_

#### Comments:

 

 

---

## Training for Clinicians and Clinical Staff

### 63) [For each of the funding streaming selected above]

**Please provide
the estimated total cost/budget expended on this intervention. If actual
expenditures are not available, budgets may be used to estimate costs. You will
have the options to provide the dollar value of the estimated cost/budget or
provide the percentage of cost/budget, or both.**

**If providing** dollar value, please round it
to the nearest $50,000. If less than $25,000, please mark as $0.:
\_\_\_\_\_\_\_\_\_\_\_\_\_\_\_\_\_\_\_\_\_\_\_\_\_\_\_\_\_\_\_\_\_\_\_\_\_\_\_\_\_\_\_\_\_\_\_\_\_

**If providing** percentages, it should be
calculated across all selected programs. The percentages should add up to 100%
across all programs and funding streams.:
\_\_\_\_\_\_\_\_\_\_\_\_\_\_\_\_\_\_\_\_\_\_\_\_\_\_\_\_\_\_\_\_\_\_\_\_\_\_\_\_\_\_\_\_\_\_\_\_\_

#### Comments:

 

**[For each of the funding streaming selected above]**

#### 64) Please indicate for [the period input for launch and end date], the funding covered:

( ) All of these years

( ) Some of these years

#### Comments:

 

 

---

## Review Responses

 

### You've reached the end of the survey. Please review your responses below. If you'd like to make any changes, please use the 'Back' button. Otherwise, click 'Submit' to finalize your responses.

 

 

### 65) If you have any further comments, please write it here.

\_\_\_\_\_\_\_\_\_\_\_\_\_\_\_\_\_\_\_\_\_\_\_\_\_\_\_\_\_\_\_\_\_\_\_\_\_\_\_\_\_\_\_\_

\_\_\_\_\_\_\_\_\_\_\_\_\_\_\_\_\_\_\_\_\_\_\_\_\_\_\_\_\_\_\_\_\_\_\_\_\_\_\_\_\_\_\_\_

\_\_\_\_\_\_\_\_\_\_\_\_\_\_\_\_\_\_\_\_\_\_\_\_\_\_\_\_\_\_\_\_\_\_\_\_\_\_\_\_\_\_\_\_

\_\_\_\_\_\_\_\_\_\_\_\_\_\_\_\_\_\_\_\_\_\_\_\_\_\_\_\_\_\_\_\_\_\_\_\_\_\_\_\_\_\_\_\_

 

---

## Thank You!

 

### Thank you for completing our survey, your response is very important to us.

 

---
